# The Lymphocytic Choriomeningitis Virus Matrix Protein PPXY Late Domain Drives the Production of Defective Interfering Particles

**DOI:** 10.1371/journal.ppat.1005501

**Published:** 2016-03-24

**Authors:** Christopher M. Ziegler, Philip Eisenhauer, Emily A. Bruce, Marion E. Weir, Benjamin R. King, Joseph P. Klaus, Dimitry N. Krementsov, David J. Shirley, Bryan A. Ballif, Jason Botten

**Affiliations:** 1 Department of Medicine, Division of Immunobiology, University of Vermont, Burlington, Vermont, United States of America; 2 Cellular, Molecular, and Biomedical Sciences Graduate Program, University of Vermont, Burlington, Vermont, United States of America; 3 Department of Biology, University of Vermont, Burlington, Vermont, United States of America; 4 Department of Microbiology and Molecular Genetics, University of Vermont, Burlington, Vermont, United States of America; Icahn School of Medicine at Mount Sinai, UNITED STATES

## Abstract

Arenaviruses cause severe diseases in humans but establish asymptomatic, lifelong infections in rodent reservoirs. Persistently-infected rodents harbor high levels of defective interfering (DI) particles, which are thought to be important for establishing persistence and mitigating virus-induced cytopathic effect. Little is known about what drives the production of DI particles. We show that neither the PPXY late domain encoded within the lymphocytic choriomeningitis virus (LCMV) matrix protein nor a functional endosomal sorting complex transport (ESCRT) pathway is absolutely required for the generation of standard infectious virus particles. In contrast, DI particle release critically requires the PPXY late domain and is ESCRT-dependent. Additionally, the terminal tyrosine in the PPXY motif is reversibly phosphorylated and our findings indicate that this posttranslational modification may regulate DI particle formation. Thus we have uncovered a new role for the PPXY late domain and a possible mechanism for its regulation.

## Author Summary

Arenaviruses cause severe and often fatal diseases in humans yet typically establish lifelong, asymptomatic infections in their rodent reservoirs. Several families of enveloped RNA viruses, including the arenaviruses, encode short amino acid motifs, called late domains, to hijack host proteins in the endosomal sorting complex required for transport (ESCRT) to drive the release of virus particles from the host cell’s outer membrane. Many late domain-containing viruses produce defective interfering (DI) particles in addition to standard, infectious virus. DI particles cannot self-replicate but interfere with the production of infectious virus and mitigate virus-induced cytopathic effect. Arenaviruses such as lymphocytic choriomeningitis virus (LCMV) generate high levels of DI particles, yet, the mechanism that drives their formation is not known. We show that LCMV’s only encoded late domain, PPXY, and a functional ESCRT pathway are critical for DI particle production, but in contrast, are not absolutely required for infectious virus production. We also demonstrate that the LCMV PPXY late domain is phosphorylated and that this modification may regulate DI particle production. In summary, we have discovered a new and unexpected role for a viral late domain in selectively driving the production of DI particles independently of standard infectious virus particles.

## Introduction

Arenaviruses are a family of rodent-borne viruses with a worldwide distribution. These viruses typically establish persistent, asymptomatic infections in rodent reservoir species [1]. In contrast, arenaviruses cause severe and often fatal diseases in humans. Several arenaviruses, including Lassa virus and Junín virus, cause hemorrhagic fever syndromes whereas infection with the prototypic arenavirus, lymphocytic choriomeningitis virus (LCMV), can lead to aseptic meningitis in immunocompetent individuals, high lethality in immunocompromised individuals, or severe birth defects in the developing fetus [2,3]. U.S. Food and Drug Administration-approved vaccines do not exist for the prevention of arenavirus infection and effective antiviral therapies have been limited to the use of ribavirin for Lassa virus [4] or immune plasma for Junín virus [5].

Arenaviruses are enveloped viruses with a single-stranded, bi-segmented RNA genome that encodes four proteins in an ambisense manner. The small (S) segment encodes the nucleoprotein (NP) and glycoprotein (GP) while the large (L) segment encodes the RNA-dependent RNA polymerase (L) and the matrix protein (Z) [6]. Arenaviruses enter cells via receptor-mediated endocytosis [7], undergo genomic replication and transcription in the cytoplasm [6], and assemble and bud new particles at the plasma membrane [8]. The Z protein, which lines the luminal side of the viral membrane, is responsible for a number of critical functions in the virus life cycle, including driving the process of viral particle assembly and budding [9]. Accordingly, Z can form virus-like particles (VLPs) in the absence of other viral proteins and is thought to be both necessary and sufficient for driving the budding process [10,11].

Several VLP-based studies indicate that Z drives viral particle release by virtue of one or more encoded viral late domain(s) (P(S/T)AP, YXXL, and/or PPXY), which can recruit proteins from the cellular endosomal sorting complex required for transport (ESCRT) pathway [10–12]. ESCRT machinery is required for most cellular membrane scission events that result in separation away from the cytosol including multivesicular body formation and cellular abscission [13–15]. Many enveloped viruses are known to hijack cellular ESCRT machinery via their late domains to complete the final membrane scission step required for virions to bud from host membranes (for review see [16]).

Viruses from diverse families, including arenaviruses, produce defective interfering (DI) particles in addition to standard, infectious virus during the normal course of infection [17]. DI particles are largely similar to standard virus particles in their appearance and viral protein content but cannot self-replicate, and interfere with the production of homologous standard virus [17]. In many cases, the primary difference between DI particles and standard virus is thought to be the presence of deletions in the viral genome [18]. With regard to LCMV, small deletions in the terminal untranslated regions of genomic and antigenomic RNAs have been observed, but it is not known whether these RNAs have interfering properties or are selectively incorporated in DI particles [19]. The interfering activity of arenavirus DI particles can be blocked by neutralizing antibodies but is maintained even after treatment with ultra-violet (UV) radiation, unlike standard particles, which are highly susceptible to both treatments [20]. Arenaviruses generate high levels of DI particles both in cell culture [21] and in host rodents [22]. It has long been postulated that arenavirus DIs are an important factor in the establishment of persistent infection [17,23,24] but a causal link between arenavirus DI particles and persistence has yet to be firmly established.

There are several outstanding questions regarding the arenavirus matrix protein, including how its functionality is regulated and how, in the context of a fully replicating virus, encoded late domains contribute to the production of standard and DI particles. Herein we demonstrate that LCMV’s sole late domain, PPXY, is not required for standard virus budding but instead is the driving force of DI particle release. Further, standard virus appears to bud independently of ESCRT machinery while DI particle release is ESCRT-dependent. Finally, we show that the LCMV PPXY motif is tyrosine phosphorylated and that this post-translational modification appears to regulate DI particle formation.

## Results

### The LCMV matrix protein is reversibly phosphorylated

**Fig 1:**
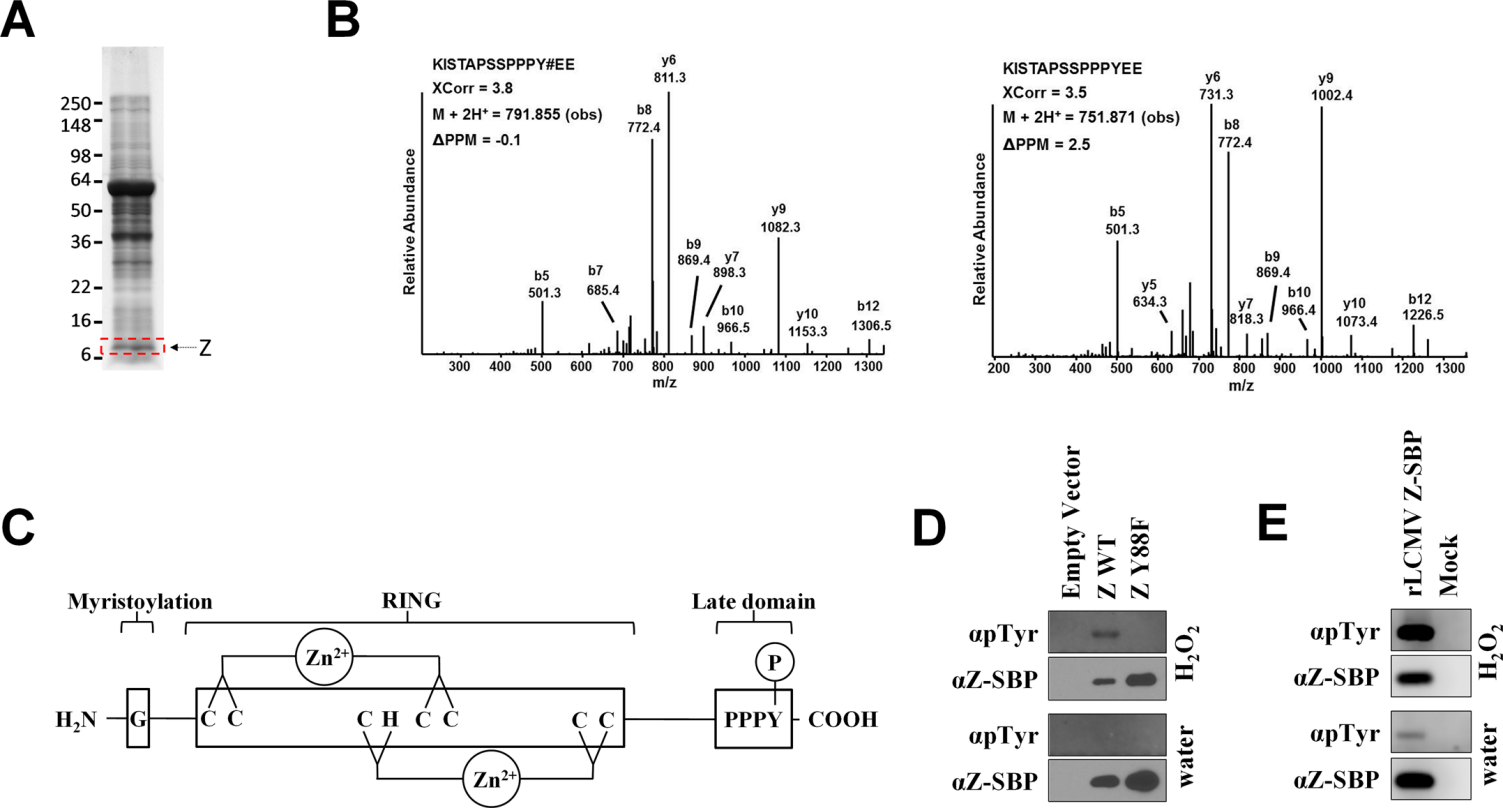
The LCMV matrix protein PPXY late domain is reversibly phosphorylated. (A) Protein lysates from sucrose-banded LCMV strain Armstrong 53b particles were separated on polyacrylamide gels and stained with Coomassie brilliant blue. A gel slice containing the Z protein (indicated by the red box) was excised, subjected to in-gel tryptic and/or chymotryptic digestion, and extracted peptides were analyzed by mass spectrometry for the presence of phosphorylated peptides as described in the Materials and Methods. (B) Representative low energy collision-induced dissociation tandem mass spectra of a chymotryptic peptide harboring the indicated phosphotyrosine residue or the same peptide unphosphorylated. Both peptides were identified from virion-derived LCMV Z protein. The tandem mass spectra were collected in an Orbitrap (MS1)-linear ion trap (MS2) mass spectrometer. Y# denotes phosphotyrosine. The SEQUEST XCorr values, the precursor observed mass and the associated PPM are indicated. S1 Fig shows the corresponding calculated and measured b- and y-type ions indicating identified fragment ion masses. (C) Depiction of the LCMV Z protein. G, glycine at position 2 that is myristoylated; RING, the central zinc-binding really interesting new gene (RING) domain; PPPY, LCMV’s only known late domain that contains the Y88 site of phosphorylation. (D) Tyrosine 88 in the LCMV matrix protein is phosphorylated. HEK293T cells were transfected with an empty vector or a plasmid encoding LCMV Z (either WT or Y88F) with a C-terminal streptavidin binding peptide (SBP) tag. Following a 15 minute exposure to either water or the tyrosine phosphatase inhibitor, H_2_O_2_, Z was affinity purified from cell lysates using magnetic streptavidin beads and screened via western blot using antibodies specific for phosphotyrosine or the SBP tag. Results are representative of 3 independent experiments. (E) LCMV is phosphorylated in rodent cells. L929 cells were infected or not with a rLCMV that encodes a streptavidin binding peptide (SBP) fusion tag at the C terminus of Z. Two days later, cells were exposed to either water or the tyrosine phosphatase inhibitor, H_2_O_2_, for 15 minutes. SBP-tagged Z was then affinity purified from cell lysates using magnetic streptavidin beads and screened via western blot using antibodies specific for phosphotyrosine or the SBP tag. Results are representative of 3 independent experiments.

The matrix protein plays a multifactorial role in the arenavirus life cycle yet little is known regarding how its various functions are regulated. Given the importance of phosphorylation for regulating the functionality of matrix proteins of other virus families [25–29], we were interested in whether LCMV’s matrix protein might also be phosphorylated. LCMV strain Armstrong 53b particles grown in Vero E6 cells were purified via sucrose-banding (Fig 1A) and subjected to mass spectrometry. This analysis revealed a tyrosine phosphorylation site near the C-terminus of the LCMV Z protein at position 88 (Y88) (Fig 1B and S1 Fig), which lies within LCMV Z’s PPPY late domain (Fig 1C). Both phosphorylated and unphosphorylated peptides containing this residue were observed at a ratio of 1 to 11, respectively, which suggests that ~10% of the total Z protein in this virion preparation is phosphorylated (Fig 1B and S1C Fig). Because the virion preparation contained a mixture of both standard infectious virus and DI particles, we were not able to determine whether the phosphorylated Z was derived from standard particles, DI particles, and/or both types of particles. To confirm the phosphorylation site, plasmids encoding either WT Z or a phenylalanine mutant (Y88F) that cannot be phosphorylated were transfected into HEK293T cells and 2 days later the cells were treated with either water or the tyrosine phosphatase inhibitor, hydrogen peroxide. WT Z and Y88F Z were affinity purified and probed with a phosphotyrosine-specific antibody. The phosphotyrosine signal detected from WT Z was greatly enhanced following inhibition of tyrosine phosphatases (Fig 1D). Substitution of tyrosine 88 with phenylalanine, to prevent phosphorylation, resulted in a complete loss of detectable phosphotyrosine signal in both settings indicating that Y88 may be the only tyrosine of the 3 encoded in LCMV Z that is phosphorylated (Fig 1D) by endogenous kinases in these cells. To determine whether LCMV Z is tyrosine phosphorylated in the context of a relevant rodent cell line, we infected murine L929 cells with a rLCMV that encodes Z with a C-terminal streptavidin binding peptide (SBP) tag. Two days later, cells were either treated with hydrogen peroxide or not and Z was affinity purified from cell lysates for western blot analysis. As shown in Fig 1E, a phosphotyrosine signal was clearly detectable from Z and was enhanced following treatment with hydrogen peroxide.

### The LCMV matrix protein PPXY late domain is dispensable for the production of standard infectious particles

**Fig 2:**
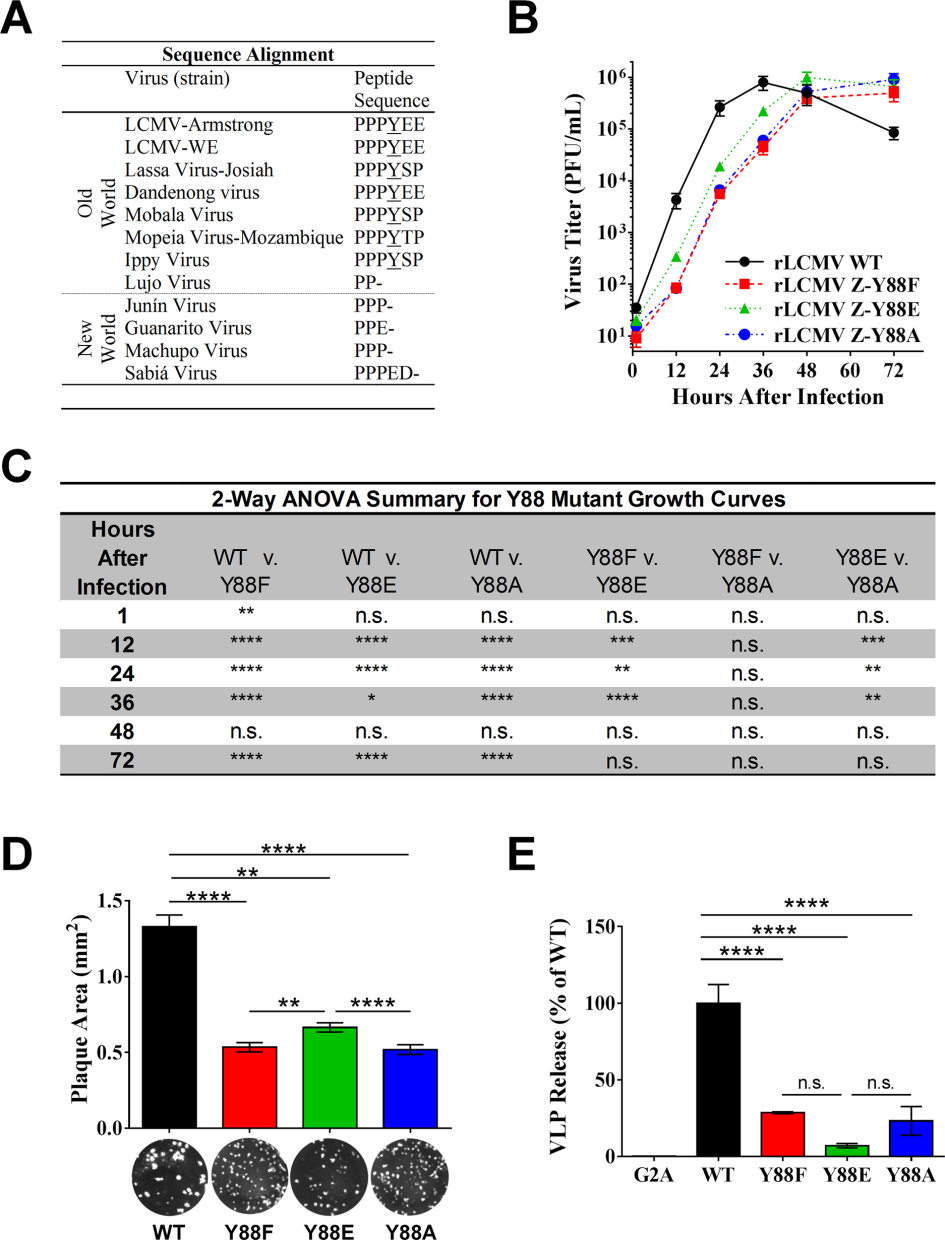
The LCMV Z PPXY late domain is dispensable for the production of infectious LCMV particles. (A) Sequence alignment of arenavirus Z proteins reveals conservation of Y88 among most Old World, but not New World, arenaviruses. (B) Recombinant (r)LCMV containing substitutions at Z Y88 that either mimic constitutive phosphorylation (Y88E) or cannot be phosphorylated (Y88F and Y88A) were generated using reverse genetics as described in the Materials and Methods. Vero E6 cells were infected at an MOI of 0.01 and the quantity of infectious virus released at each of the indicated time points was determined via plaque assay. Data are presented as mean PFU ± standard error of the mean (SEM) of 3 independent experiments. (C) Summary of two way analysis of variance (ANOVA) with Holm-Sidak’s test for multiple comparisons of log-transformed data for virus growth curves in (B). (D) The area of plaques from rLCMV WT or Z Y88 mutants was measured using Image J. Data represent the mean ± SEM of plaques analyzed from 16 wells from 6-well plates. Mean values were compared using the Kruskal-Wallis non-parametric test with Dunn’s multiple comparisons test. (E) Mutation of the PPXY domain reduces Z budding function in a VLP assay while phosphorylation of this domain at Y88 does not further impact budding. A plasmid encoding LCMV Z WT, Z G2A, or the indicated Z Y88 mutants was transfected into HEK293T cells and 1 day later cells and VLP-containing supernatants were collected and screened via quantitative western blot for Z. The percent VLP release was calculated as the amount of Z protein found in the cell culture media relative to the amount in cells. Data are presented as mean release ± SEM relative to WT Z from 3 independent experiments. A one way ANOVA with Holm-Sidak’s test for multiple comparisons was used to compare the mean values. (C-E), n.s. (not significant), *p < 0.05; **p < 0.01; ***p < 0.001; ****p < 0.0001, as determined by the indicated statistical tests.

The finding that LCMV Z is phosphorylated at Y88 was intriguing as this residue is part of LCMV’s only late domain, PPPY. This motif is well conserved among most Old World arenavirus Z proteins (Fig 2A) and its importance for the budding activity of LCMV and Lassa virus Z in the context of VLP-budding assays has been well described [10,11]. To investigate the role of this late domain in the context of authentic virus and to determine whether tyrosine phosphorylation may regulate its function, we generated recombinant (r)LCMV encoding either phenylalanine or alanine at position 88 to prevent phosphorylation at this site or glutamic acid to mimic constitutive phosphorylation. The alanine mutant was included as a reference to previous studies on the function of this late domain for LCMV and Lassa virus Z, which used alanine substitutions at Y88 to assess the contribution of this late domain to Z’s budding efficiency in VLP assays [10,11]. Viruses containing all three mutations were recoverable despite the well-described defect in Z’s budding efficiency caused by mutation of this residue (Fig 2B) [10,11]. The growth kinetics of rLCMV Z-Y88F and Z-Y88A during the first 36 hours (hr) post-infection (pi) were nearly identical, but impaired ~15-fold compared to rLCMV WT (P ≤ 0.0001; Fig 2B and 2C). The growth rate of the rLCMV Z-Y88E phosphomimetic was also attenuated compared to WT virus over this same time frame (~4-fold less PFU at 36 hr pi, P ≤ 0.05, Fig 2B and 2C). However, the phosphomimetic virus grew to ~4–fold higher titers than the alanine or phenylalanine mutants (P ≤ 0.01; Fig 2B and 2C). Additionally, the mean plaque size for rLCMV Z-Y88E was significantly increased compared to the Z-Y88F and Z-Y88A viruses (0.67 vs 0.53 or 0.52 mm^2^; P ≤ 0.01; Fig 2D), indicating that virus spread was partially restored in the phosphomimetic virus. Notably, each mutant virus eventually reached peak WT titers. Given the delayed kinetics observed in the mutant viruses, we tested for reversion mutations at 72 hr pi and confirmed that each virus retained its respective mutated residue at position 88 and its small plaque phenotype (Fig 2D). Collectively, these results demonstrate that the PPXY late domain is not absolutely required for the formation and release of standard infectious particles. Further, phosphorylation of Y88 may have a positive regulatory impact on viral propagation.

### Phosphorylation of the PPXY late domain does not enhance Z’s ability to form VLPs

Point mutations made at Y88 suggested that dynamic phosphorylation of this residue was important for the function of the matrix protein. Given the important role of the LCMV matrix protein and its late domain motif in regulating viral budding [10,11], we next investigated the specific effect these point mutations had on Z’s budding efficiency in a VLP release assay. Because the LCMV Z protein is sufficient for the production of VLPs in the absence of any other viral proteins [10,11], we were able to assess the budding activity of plasmid-derived WT or Y88-mutant Z proteins. As a control, we also included the LCMV Z G2A mutant, which exhibits a pronounced defect in VLP formation due to its inability to be myristoylated at this glycine residue [30]. HEK293T cells were transfected with plasmids encoding WT or Y88 mutants and 1 day later the VLP-containing supernatant and cells were collected and analyzed by quantitative western blotting. The budding activity of all three Z Y88 mutants was significantly reduced compared to WT Z, indicating that mutations in this region reduce the efficiency of VLP release (Fig 2E). In particular, the impaired VLP release exhibited by the Z Y88A mutant confirmed earlier findings by Perez et al. [11]. We did not observe a significant difference between the budding of the Z-Y88E phosphomimetic compared to Y88F and Y88A (Fig 2E). This suggests that the partial gain of fitness observed with the phosphomimetic rLCMV-Z-Y88E virus in Fig 2B is not due to an increase in budding activity and as such Y88 phosphorylation does not appear to directly regulate the budding function of this late domain. However, because the VLP budding assay measures only the release of matrix protein, in the absence of other viral proteins, it is possible that this assay does not recapitulate all the facets of infectious virion production.

### PPXY late domain mutant viruses release substantially less viral structural proteins and genomes without a corresponding loss of infectious units

**Fig 3:**
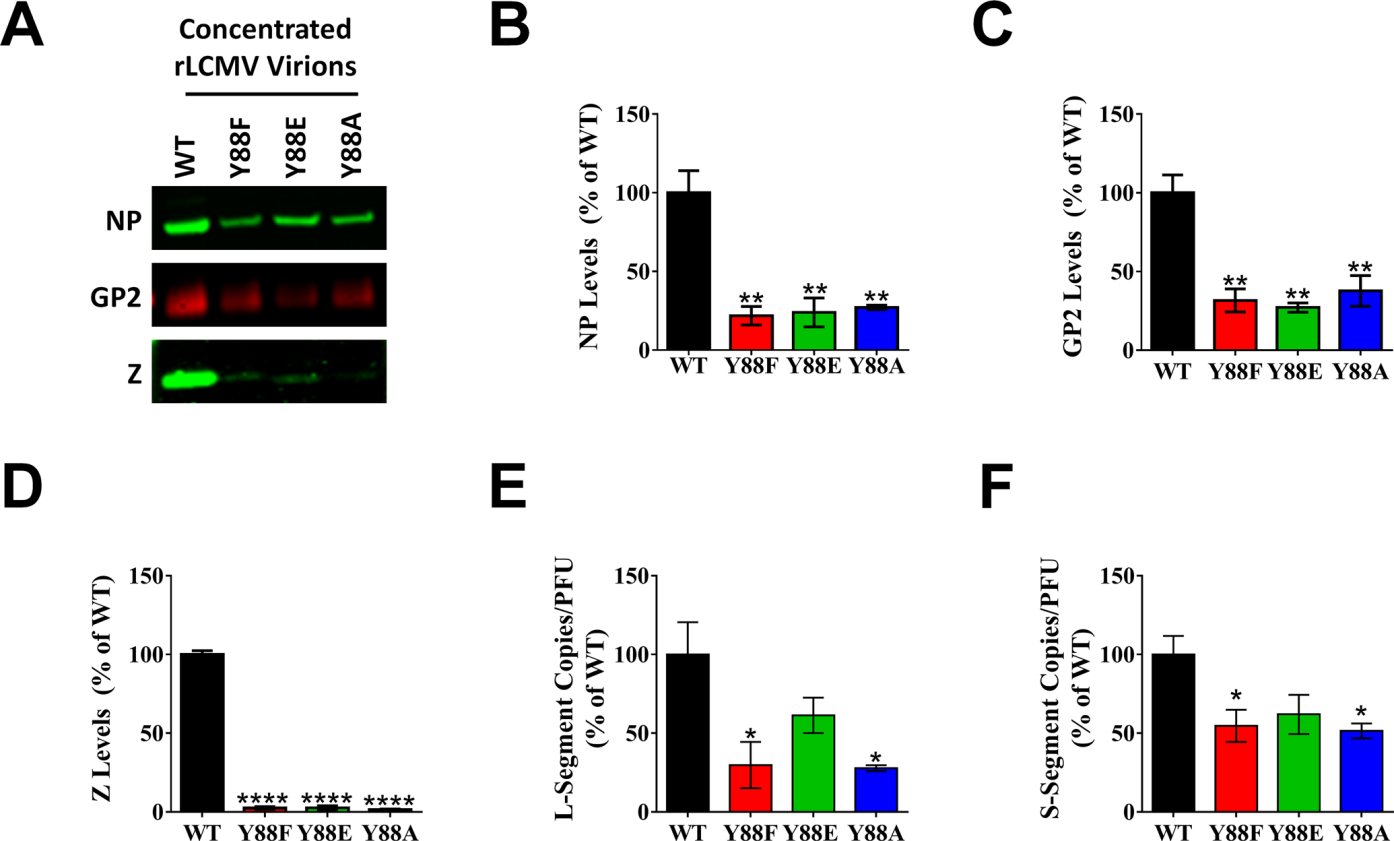
PPXY late domain mutant viruses release substantially less viral structural proteins and genomes without a corresponding loss of infectious units. (A-E) An equal quantity of PFUs of rLCMV WT, Z Y88F, Z Y88E, or Z Y88A were concentrated via ultracentrifugation through sucrose and screened for viral NP (B), GP2 (C), or Z (D) via quantitative western blot or L segment vRNA (E) or S segment vRNA (F) via qRT-PCR. Representative western blots for NP, GP2, and Z are shown in (A). Data in (B-F) are representative of the mean ± SEM relative to rLCMV WT from at least 3 independent experiments. (B-E) *p < 0.05; **p < 0.01; ****p < 0.0001, determined using the one-way ANOVA with Holm-Sidak’s test for multiple comparisons. Note that in panels B-F, the Y88 mutant viruses were not statistically different from one another.

To investigate the protein and genome composition of virions containing mutated late domains, an equivalent quantity of cell-free infectious virus particles from each rLCMV strain was concentrated for screening. Quantitative western blotting revealed substantial reductions in the total amount of NP, GP, and Z in the Y88 mutant particles relative to WT virus (Fig 3A–3D). However, no difference was observed in the levels of these proteins among the three Y88 mutant viruses (Fig 3A–3D). The quantity of Z protein detected in the Y88 mutant virus preparations was <3% of WT virus (Fig 3D) whereas NP and GP quantities were ~25% of WT virus (Fig 3B and 3C). Viral genome content in particles was assessed by qRT-PCR. On a per PFU basis, the quantity of either L or S segment genomic RNA in the non-phosphorylatable mutants, Y88F and Y88A, was significantly reduced versus WT (P ≤ 0.05, Fig 3E and 3F). However, genome levels in the phosphomimetic virus, Y88E, were not significantly different than WT (Fig 3E and 3F), which may explain a component of its partially restored growth kinetics (Fig 2B). The observation of reduced viral proteins and/or genomes released from cells infected with the Y88 mutant viruses combined with the fact that WT LCMV is known to produce relatively large quantities of DI particles [21] led us to hypothesize that the PPXY mutants may have defects in their ability to generate DI particles, which could explain their greatly reduced levels of viral protein and genome relative to PFU.

### The PPXY late domain drives the production of DI particles

**Fig 4:**
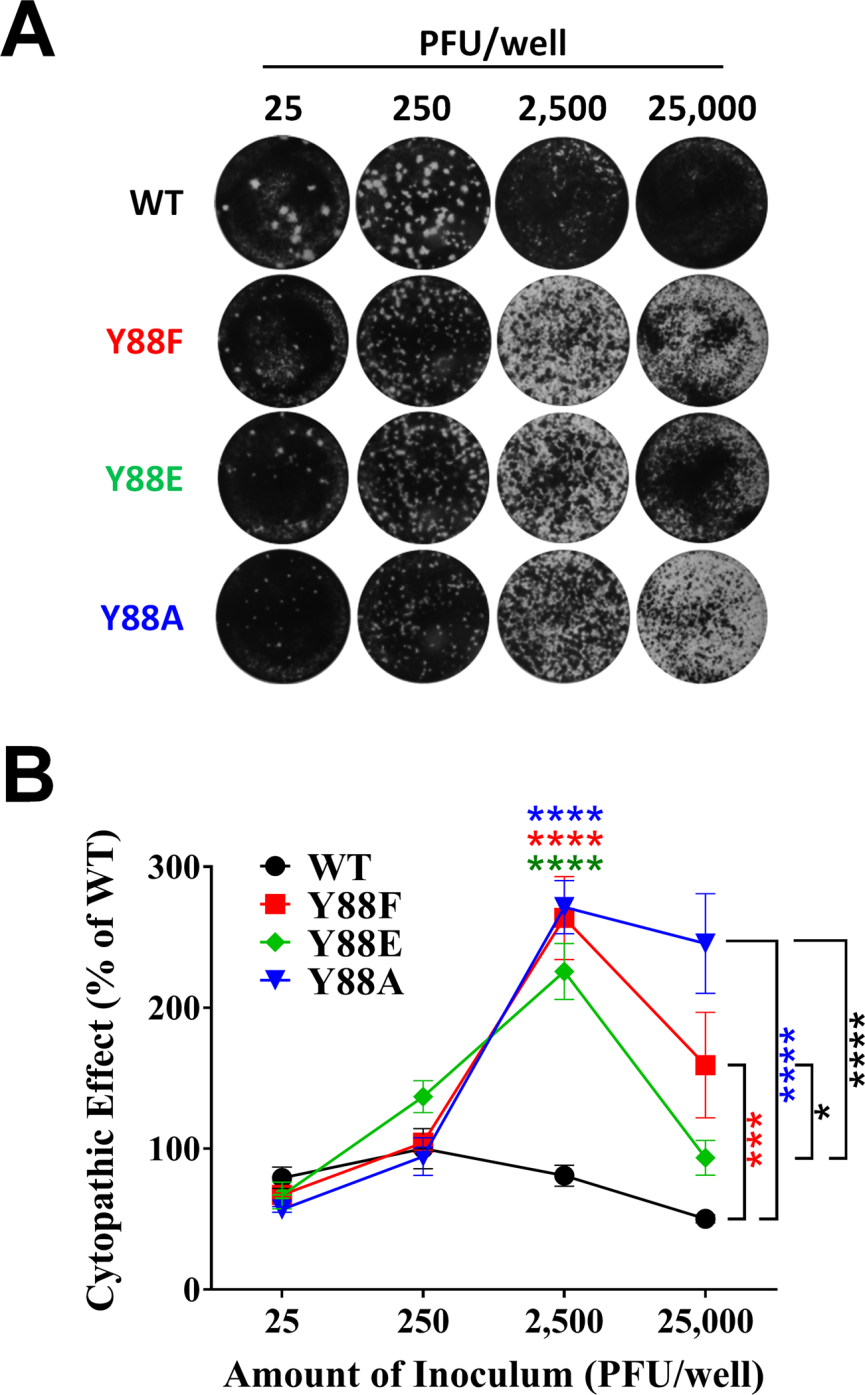
LCMV DI particle production is impaired in the absence of a functional PPXY domain. (A-B) Equivalent PFUs of WT, Z Y88F, Z Y88E, or Z Y88A rLCMV (range 25 to 2.5 x 10^4^ PFUs) were inoculated onto monolayers of Vero E6 cells and a standard plaque assay was performed. Representative images of crystal violet-stained wells are shown in (A). Inhibition of standard infectious virus-induced cytopathic effect by DI particles at each dose was determined by measurement of the mean pixel intensity of each well using Image J software (B). The data in (B) are representative of the mean ± SEM relative to rLCMV WT (at 250 PFU per well) from 3 independent experiments and were tested for statistical significance with a two way ANOVA and Holm-Sidak’s test for multiple comparisons. (B) *p < 0.05; ***p < 0.001; ****p < 0.0001, as determined by the indicated statistical tests.

A substantial fraction of virus particles produced by LCMV are DI particles [31]. Accordingly, inoculation of LCMV at low multiplicities of infection (MOI) results in efficient production of standard virus and spread, while high MOIs do not. This seemingly contradictory phenomenon is caused by DI particles, which inhibit the propagation of standard virus and its ability to cause cytopathic effect with one hit kinetics [21,32]. Monolayers inoculated with high concentrations of standard infectious LCMV exhibit no cytopathic effect due to DI particle inhibition, but as the inoculum is diluted, standard virus particles that infect cells in the absence of a co-infecting DI particle will subsequently form plaques. We exploited this phenomenon to initially evaluate the relative amounts of DI particles generated by the PPXY mutant viruses. Equal infectious doses of WT virus and each Y88 mutant, spanning a range of 25 to 25,000 PFU, were applied to Vero E6 cell monolayers in a standard plaque assay (Fig 4A and 4B). Evidence of possible DI particle interference is clearly seen in WT virus, where the most concentrated viral sample (25,000 PFU) resulted in no cell death while in successive 10-fold dilutions (2,500 and 250 PFU) the number of DI particles per cell is lowered allowing standard virus to enter cells in the absence of DI particles and form plaques (Fig 4A). In contrast, the PPXY-mutant viruses exhibited a considerable increase in cytopathology (Fig 4A, 25,000 and 2,500 PFU). Quantification of the observed cytopathology confirmed the striking phenotype and revealed significant differences between the mutant and WT viruses (Fig 4B). Intriguingly, the cytopathology of the rLCMV Z-Y88E phosphomimetic at 25,000 PFU was significantly less than both Y88F or Y88A viruses and therefore more closely resembled WT virus (Fig 4B).

**Fig 5:**
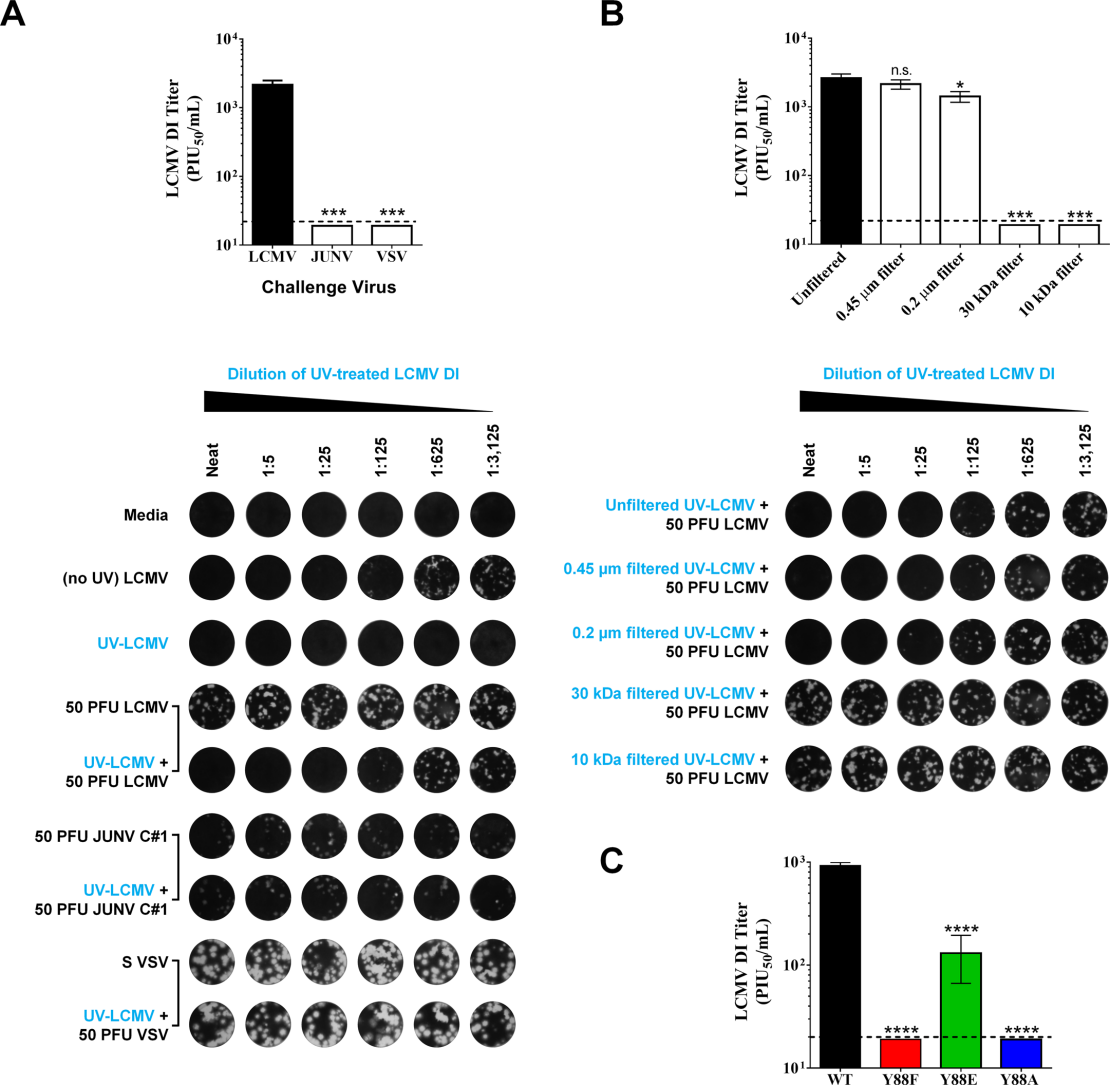
The PPXY late domain drives the production of DI particles. (A-B) Development and validation of a plaque interference assay for the measurement of LCMV DI particle activity. In (A), a stock of rLCMV WT containing both standard infectious virus particles and DI particles was subjected to UV irradiation for 2 min to inactivate standard LCMV particles but spare DI particles. Serial 5-fold dilutions of this UV-treated virus preparation (UV-LCMV) were added to Vero E6 cells followed by a fixed amount of 50 PFU of the indicated challenge virus (rLCMV WT; Junín virus Candid 1, (JUNV C#1), or vesicular stomatitis virus (VSV)). Additional controls were wells that received i) media only, ii) serial 5-fold dilutions of the stock LCMV virus preparation before UV treatment (no UV LCMV), iii) serial 5-fold dilutions of the stock LCMV virus preparation following UV treatment (UV-LCMV), or iv) 50 PFU per well of standard LCMV, JUNV C#1, or VSV, as indicated. Following a 1 hr incubation at 37°C to permit viral particle absorption, cells were overlaid with agarose and subsequently fixed and stained with crystal violet to visualize whether the UV-LCMV preparation impacted the ability of each virus to form plaques. The LCMV DI titer is expressed as plaque interfering units_50_ (PIU_50_) per mL of a given sample and was calculated as described in the Materials and Methods. In (B), rLCMV WT containing both standard and DI particles was subjected to the indicated filtration or not and then subjected to UV irradiation as described in (A). Serial 5-fold dilutions of each UV-LCMV preparation (filtered or not) were added to Vero E6 cells followed by a fixed amount of 50 PFU of rLCMV WT. As described in (A), the ability of these UV-LCMV preparations to interfere with the ability of standard LCMV to from plaques was measured via plaque assay and LCMV DI titers are reported as PIU_50_/mL. In (A-B), the graphical results represent the mean LCMV DI titer ± SEM for 3 independent experiments and representative wells for each condition are shown directly below each graph. (C) LCMV DI particle production requires a functional PPXY domain. The rLCMV WT or Y88 mutants examined in Fig 4 were subjected to the assay described in (A-B) to directly measure the DI particle titer present in each virus preparation. Briefly, each indicated rLCMV preparation was subjected to UV-irradiation to inactivate standard infectious LCMV particles while preserving DI particle activity. Serial 5-fold dilutions of each UV-treated sample were inoculated onto Vero E6 cells, followed by the addition of 50 PFU of standard LCMV. Following a 1 hr incubation at 37°C to permit viral particle absorption, cells were overlaid with agarose and subsequently fixed and stained with crystal violet to visualize whether the various UV-treated rLCMV preparations impacted the ability of standard LCMV particles to form plaques. For each rLCMV, DI titer is reported as mean PIU_50_/mL ± SEM for 3 independent experiments. (A-C) n.s. (not significant), *p < 0.05; ***p < 0.001; ****p < 0.0001, determined by first substituting values of 19 PIU_50_/mL (just below the limit of detection value of 20 PIU_50_/mL) for samples that were below the limit of detection and then performing a one way ANOVA.

To confirm that the interfering activity observed in Fig 4 was indeed due to LCMV DI particles, we next established an assay to directly and quantitatively measure LCMV DI particle activity. At present, no consistent biochemical or genetic signature exists to distinguish LCMV DI particles from standard infectious particles [33,34]. In an attempt to uncover such a signature, we separated preparations of rLCMV WT or Y88 mutants via density ultracentrifugation. Similar to previous studies [33–37], we were unable to isolate fractions containing pure DI particles as abundant levels of standard virus were detectable across all 15 fractions (S2 Fig). Therefore, it was not possible to identify a DI particle-specific signature for screening purposes. Despite this limitation, several assays, including a yield reduction assay [21], a plaque reduction assay [38], and a focus interfering assay [32] have historically been used for accurate measurement of LCMV DI particle abundance and activity levels. Indeed, these assays were originally used to define LCMV DI particles. We utilized the plaque interference assay (also known as the plaque reduction assay) analogous to that used in [21] but also capitalized on the strong UV-resistance exhibited by LCMV DI particles, but not standard virus particles [38]. Briefly, cell-free virus preparations containing both standard and DI particles were treated with UV to neutralize standard virus particles while leaving the interfering properties of DI particles intact (Fig 5A). It should be noted that standard virus particles treated with UV do not acquire detectable interfering properties (Fig 5A) [39]. Limiting dilutions of this UV-treated sample were applied to Vero E6 cells, followed by the addition of a fixed quantity of LCMV PFUs. As shown in Fig 5A, this allows for the determination of LCMV DI particle activity and is expressed as plaque interfering units_50_ (PIU_50_) per mL of a given sample. Importantly, we recapitulated several key controls from previous studies to demonstrate the specificity of this assay for LCMV DI particles. In particular, UV-treated LCMV DI particle preparations only interfered, in a dose-dependent manner, with the growth of homologous LCMV, but not heterologous viruses such as vesicular stomatitis virus (VSV) or the New World arenavirus Junín virus Candid 1 (JUNV C#1), which rules out a nonspecific antiviral factor as a mediator of interference (e.g. interferon) (Fig 5A). Further, passing LCMV DI particle-containing supernatant through a series of filters (0.45 μm, 0.2 μm, 30 kDa, 10 kDa) showed that interference is not due to soluble factors that are smaller than 30 kDa (e.g. cytokines) or larger (>0.2 μm) membrane bound entities such as bacteria (Fig 5B). When this assay was applied to the rLCMV WT and Y88 mutant samples examined in Fig 4, it confirmed that the rLCMV WT samples exhibited substantial DI particle interfering activity (mean 926 PIU_50_/mL ± 68 SEM), but that the mutant Y88 viruses had much less (Fig 5C). There was no detectable DI activity for either the Y88F or Y88A viruses while the Y88E virus contained intermediate levels of interfering activity (mean 131 PIU_50_/mL ± 64 SEM). Collectively, the findings in Figs 4 and 5 support the hypothesis that the LCMV PPXY late domain is required for the efficient formation of DI particles and that phosphorylation of Y88 may play a regulatory role in DI particle production and the inhibition of cytopathic effect.

### Efficient DI particle formation requires a functional ESCRT pathway

**Fig 6:**
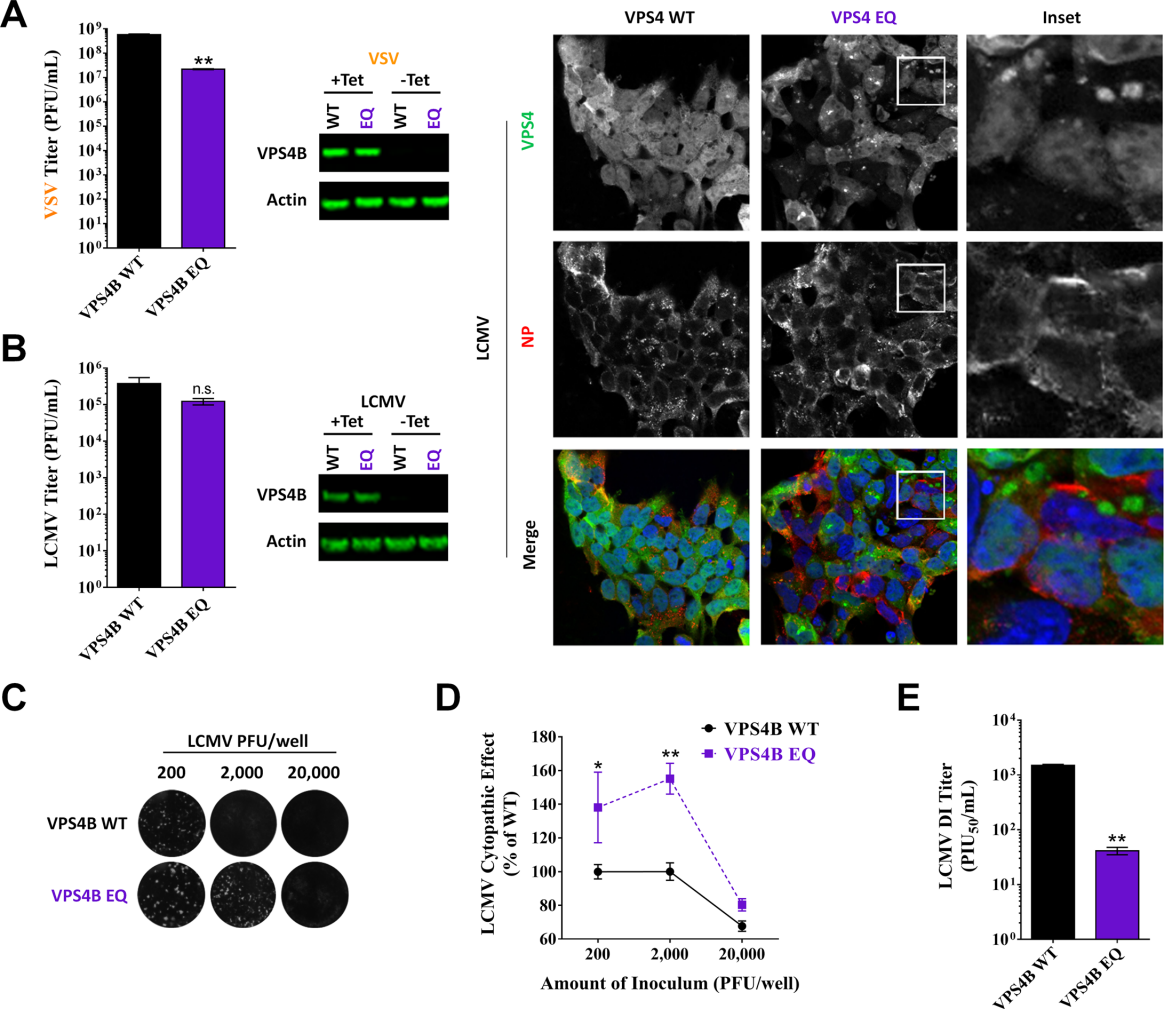
Efficient DI particle formation requires a functional ESCRT pathway. (A) VSV requires a functional ESCRT pathway for infectious virus release. T-Rex HEK293 cells stably transduced with vectors for tetracycline-based induction of WT vacuolar protein sorting 4B (VPS4B) or the DN VPS4B mutant, EQ, were treated with tetracycline to induce the expression of WT or DN VPS4B. Both the WT and DN VPS4B proteins have GFP fusion tags. One hr following VPS4B induction, the media was removed and cells were infected with VSV in media containing tetracycline. One hr later, the viral inoculum was removed and replaced with fresh media containing tetracycline. Six hr later (8 hr post-VPS4B induction; 7 hr post-infection), supernatants were collected to determine VSV PFU titers via plaque assay. Cell protein lysates were also generated at this time to verify the induction of VPS4B WT or EQ expression using an anti-GFP antibody. Protein lysates were also screened for actin as a loading control. Viral titers represent the mean VSV PFU ± SEM from 3 independent experiments and were tested for statistical significance with an unpaired t test with Welch’s correction. (B-E) LCMV requires a functional ESCRT pathway for the release of DI particles, but not standard infectious particles. T-Rex HEK293 cells stably transduced with vectors for tetracycline-based induction of WT VPS4B or the DN VPS4B EQ were infected with rLCMV WT and 2 d later treated with tetracycline to induce the expression of WT or DN VPS4B. Six hr after VPS4B induction (54 hr pi), the cells were washed and given fresh media containing tetracycline. Supernatants were collected 18 hr later (72 hr pi) and titered via plaque assay. Similar to (A), protein lysates were collected at 72 hr pi and screened for VPS4B WT or DN using an anti-GFP antibody or for actin as a loading control. Extra wells containing cells grown on cover slips were also fixed at 72 hr pi to examine, via immunofluorescent confocal microscopy, the expression and localization of WT or DN VPS4B (green) or LCMV NP (red). A 143 μm square is shown for each panel. The results shown in (B) represent the mean LCMV PFU ± SEM from 4 independent experiments and were tested for statistical significance with an unpaired t test with Welch’s correction. (C) Equivalent PFUs of virus (range 2 x 10^2^ to 2 x 10^4^) produced from WT or DN VPS4B cells were inoculated onto monolayers of Vero E6 cells and a standard plaque assay was performed. Representative images of crystal violet-stained wells are shown in (C). Inhibition of standard infectious virus-induced cytopathic effect by DI particles at each dose was determined in (D) by measurement of the mean pixel intensity of each well using Image J software. The data in (D) are representative of the mean ± SEM relative to WT VSP4B (at 2,000 PFU per well) from 4 independent experiments and were tested for statistical significance with a two way ANOVA and Holm-Sidak’s test for multiple comparisons. (E) The assay described in Fig 5 was used to directly measure the LCMV DI titer present in the supernatants collected from the VSP4B WT or DN cells at 72 hr pi. Briefly, each virus-containing preparation was subjected to UV-irradiation to inactivate standard infectious LCMV particles while preserving DI particle activity. Serial 5-fold dilutions of each UV-treated sample were inoculated onto Vero E6 cells, followed by the addition of 50 PFU of standard LCMV. Following a 1 hr incubation at 37°C to permit viral particle absorption, cells were overlaid with agarose and subsequently fixed and stained with crystal violet to visualize whether the various UV-treated rLCMV preparations impacted the ability of standard LCMV particles to form plaques. The LCMV DI titers are reported as mean PIU_50_/mL ± SEM for 4 independent experiments and were tested for statistical significance with an unpaired t test with Welch’s correction. (A-B, D-E) n.s. (not significant), *p < 0.05, **p < 0.01, as determined by the indicated statistical tests.

Viral late domains can drive virus budding by recruiting components of the cellular ESCRT pathway to complete the final membrane scission step. Given the important role that the LCMV PPXY late domain played in the production of DI particles (Figs 4A, 4B and 5C), we hypothesized that this late domain might be recruiting the ESCRT pathway machinery to drive DI particle formation. To test this hypothesis, we utilized cell lines that lack a functional ESCRT pathway due to inducible expression of a dominant negative (DN), E235Q point mutant, of VPS4, an ATPase involved in the final stages of ESCRT pathway function [40–42]. Because the ESCRT pathway can also affect LCMV entry [43], we first infected cells with LCMV for 48 hr to allow the entire monolayer to become infected before inducing expression of WT or DN VPS4. The cells were washed and fresh media containing the induction agent was added to the cells 6 hr after initial induction (54 hr pi) and the virus-containing media was collected 18 hr later (72 hr pi) to determine levels of standard infectious particles and DI particles (Fig 6). Western blot analysis of protein lysates at 72 hr pi confirmed the strong induction of WT and DN VPS4B expression and examination of fixed coverslips showed that all cells were expressing both the induced VSP4B as well as LCMV NP (Fig 6B). This infection protocol was chosen to ensure that we were examining virus that was produced in cells expressing the induced VPS4B proteins, while minimizing the effect that these proteins could exert on viral entry. Expression of DN VPS4B had no impact on the release of standard infectious LCMV (P = 0.27; Fig 6B). In contrast, expression of DN VPS4B led to a marked reduction in the release of infectious VSV particles (Fig 6A), which is consistent with previous studies [42] and confirms the specificity of our findings for LCMV. Measuring LCMV DI particle activity as described in Fig 4 revealed that WT LCMV produced considerably fewer DI particles per standard infectious virus particle in the DN VPS4B background when compared to cells expressing WT VPS4B (Fig 6C and 6D). A similar trend for both LCMV infectious virus and DI particle activity was seen in cells expressing WT or DN VPS4A (S3 Fig). We next used the assay described in Fig 5 to directly quantitate the LCMV DI particle activity in these samples. Consistent with the findings in Fig 6C and 6D, this demonstrated that significantly fewer DI particles are made in the context of the DN VPS4B background when compared to WT VPS4B (mean 41 ± 6 SEM vs 1,491 ± 70 PIU_50_/mL, respectively; P = 0.0022). Thus it appears that LCMV DI particle formation requires a functional ESCRT pathway (Fig 6C–6E) in addition to a canonical late domain (Figs 4 and 5C) while standard particles do not (Fig 6B).

## Discussion

**Fig 7:**
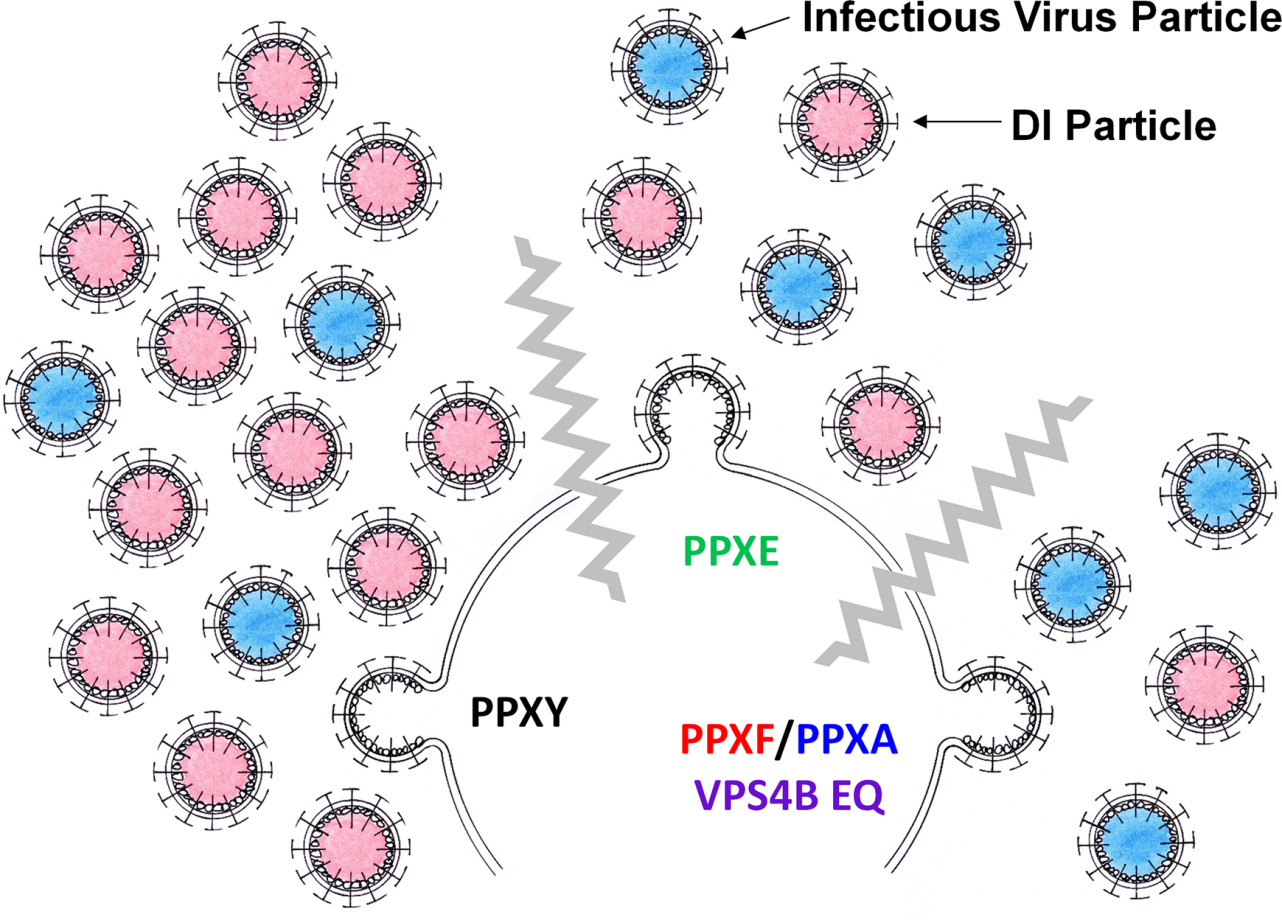
Proposed model of PPXY-driven DI particle production. WT virus containing an intact PPXY late domain produces high levels of infectious and DI particles. Disruption of the PPXY motif (PPXF or PPXA) or disruption of the ESCRT pathway causes decreased overall DI particle production compared to standard infectious particles. The phosphomimetic PPXE virus has an intermediate phenotype.

The ability of most arenavirus matrix proteins to drive viral budding is thought to be highly dependent upon one or more encoded late domains [10,11]. The arenavirus LCMV encodes a single late domain, PPPY. The PPXY motif is found in the matrix proteins of several families of enveloped RNA viruses and for many of these viruses is required for the release of infectious virions in an ESCRT-dependent fashion (for review see [16]). We demonstrate here that the PPXY late domain encoded by LCMV is not absolutely required for infectious virus release. Further, our data suggest that infectious particle release can occur in the absence of a functional ESCRT pathway. Strikingly, we show that the formation of LCMV DI particles critically requires a functional PPXY late domain and that this process is ESCRT-dependent (see Fig 7 for our proposed model). Last, our data demonstrate that the terminal tyrosine in the LCMV PPXY motif is phosphorylated and that this posttranslational modification may exert a regulatory effect on Z’s ability to drive DI particle release. Therefore, we have uncovered an unexpected role for the PPXY late domain and a possible mechanism for its regulation of DI particle production.

Our findings raise the intriguing possibility that LCMV utilizes divergent pathways for the production of infectious and DI particles. Neither a functional PPXY motif nor ESCRT pathway were absolutely required for the release of standard infectious particles. These findings, combined with the fact that LCMV Z does not encode additional canonical late domains, strongly suggest that infectious LCMV release occurs through a novel, unknown mechanism. While the rLCMV PPXY mutants studied here initially displayed a slight lag in infectious virus release, each ultimately matched WT levels. Consistent with an earlier study by Perez et al. [11], we observed that mutation of the PPXY domain impairs the ability of LCMV Z to form VLPs (Fig 2E). This finding in the VLP system may accurately reflect the initial lag in infectious virus release seen for rLCMVs bearing the same PPXY mutations (Fig 2B and 2C) and/or their decreased ability to form DI particles (Figs 4 and 5). With regard to the importance of the ESCRT pathway for infectious virus production, expression of DN VPS4B had no impact on the ability of WT rLCMV to form standard infectious virus (Fig 6B). Similarly, expression of DN VPS4B did not impair the ability of LCMV Z to form VLPs when compared to cells expressing WT VPS4B (see S5 Fig). This VLP-based result does not agree with an earlier finding by Perez et al. whereby silencing expression of the ESCRT component TSG101 impaired the release of infectious LCMV VLPs [11]. This discrepancy may reflect differences in the particular VLP assays employed (VLP release versus infectious VLP release and transduction) or perhaps the format of the experiments (siRNA silencing of TSG101 versus inducible expression of WT or DN VPS4B). The different VLP systems could also recapitulate different aspects of virus particle release, with one VLP assay perhaps mimicking infectious virus production while the other more closely resembles DI formation. In similar experiments featuring rhabdoviruses [44,45], Ebola virus [46], retroviruses [47–50], and Hepatitis B virus [51], loss of the PPXY motif and/or ESCRT resulted in standard virus growth that remained attenuated compared to WT. Interestingly, the New World arenavirus Pichinde remains attenuated following disruption of its encoded late domain (PSAP) [52], while the matrix protein encoded by the New World arenavirus Tacaribe can form VLPs in the absence of its late domain (YXXL), but requires VPS4 [53]. These findings suggest that arenaviruses have evolved diverse strategies to drive infectious virus release. One possible explanation for the observed late domain-independent generation of infectious LCMV particles is that the LCMV Z protein, either by itself or in combination with other viral structural proteins, may be sufficient to drive particle release in the absence of recruited host proteins. Alternatively, LCMV may contain additional sequence motifs, either in Z or the other structural proteins, that recruit novel host protein machinery to facilitate budding.

In contrast to standard virus particles, both the PPXY late domain and the ESCRT pathway appear critical for the release of DI particles. To our knowledge, this is the first example of a virus utilizing a late domain to selectively drive the production of DI particles independently of standard virus. That LCMV has evolved such a mechanism likely reflects the presumed importance of DI particles for the successful establishment of an asymptomatic, persistent infection in reservoir rodents, which ultimately ensures the long term maintenance of LCMV in nature [19,22]. While the existence of arenavirus DI particles has long been realized, surprisingly little is known about their exact composition and properties. Our data demonstrates that cells infected with the rLCMV PPXY mutants release much less NP, GP, and Z per PFU of cell-free virus when compared to WT rLCMV. This is presumably due to reduced levels of DI particles being released by the PPXY mutant viruses. Interestingly, the degree of reduction was not equivalent among the viral proteins. In particular, Z was reduced to the greatest extent (~3% of WT) when compared to NP or GP (~25% of WT), which could indicate that Z itself is enriched in DI particles and is critical for the ability of DI particles to interfere with the propagation of standard virus particles. Consistent with this idea, Z is able to render cells refractory to superinfection with homologous virus and treatment of DI particles with RNA-damaging levels of UV does not reduce their interfering ability [38,54]. Therefore, it is possible that particles containing high quantities of Z, or simply VLPs consisting primarily of Z, may represent a class of arenavirus DI particles.

Arenavirus matrix proteins exhibit significant diversity in the type and number of late domains they encode. The PPXY domain is found in several Old World arenavirus matrix proteins but not in New World arenaviruses (Fig 2A and [9,55]). We have observed that the New World arenavirus JUNV C#1, which encodes both a PTAP and YXXL late domain, generates considerably fewer DI particles per standard infectious particle when compared to the PPXY-containing LCMV (S4 Fig). While it is not known whether the PTAP and/or YXXL motifs contribute to DI particle formation in the case of JUNV C#1, this observation may indicate that the PPXY domain is particularly strong in driving DI particle assembly and release. It is possible that individual arenaviruses require different rates of DI particle formation for optimal fitness and have evolved to encode particular late domain combinations to best meet those needs.

We show that the LCMV Z protein is phosphorylated, which suggests that phosphorylation may be important for the regulation of one or more of Z’s functions. The fact that this modification occurs at the terminal tyrosine of the PPXY late domain and can be detected in virion-derived Z led us to hypothesize that it may influence Z’s budding function. To study the impact of this modification we generated rLCMV with mutations at tyrosine 88 that either prevented phosphorylation (Y88F or Y88A) or mimicked it (Y88E). Relative to the mutants that cannot be phosphorylated, the Y88E phosphomimetic virus generated significantly more DI particles per infectious particle (Figs 4 and 5C). This suggests that reversible phosphorylation of the PPXY motif may act as a rheostat to regulate the rate of DI particle production independent of standard virus, possibly through the recruitment of ESCRT machinery. Interestingly, the terminal tyrosine of the PPXY late domains encoded by Ebola virus and Marburg virus VP40 is also phosphorylated. Inhibiting phosphorylation of the Ebola virus VP40 PPXY motif reduced the release of infectious virus [25] whereas mutation of the Marburg virus VP40 PPXY motif to prevent phosphorylation did not impact infectious VLP release, but instead impaired the recruitment and incorporation of nucleocapsids into VLPs [29]. The role of the PPXY domain and/or its phosphorylation with regard to DI particle production in the filovirus model and other PPXY-containing virus families remains an open question. In the current model, it will be important to identify the host kinase responsible for phosphorylating the LCMV PPXY domain, although this may require a comprehensive tyrosine kinase screen as the flanking residues surrounding Y88 were not recognized by kinase motif prediction tools [56,57].

How does the PPXY late domain of LCMV promote the release of DI particles? While it is well known that the PPXY motif can drive viral budding, the exact mechanism by which it recruits ESCRT proteins to promote this process is not fully understood. Several PPXY-dependent viruses also require NEDD4 family E3 ubiquitin ligases (e.g., NEDD4, ITCH, or WWP1), which can directly bind to the PPXY motif via their WW domains (for review see [16]). Recruitment of ESCRT machinery could occur due to ubiquitination of Z by a NEDD4 E3 ubiquitin ligase, which in turn could recruit ESCRT components (e.g. TSG101) that can bind ubiquitinated proteins [16]. Alternatively, arrestin-related trafficking adaptors (ARTs) may provide an ESCRT linkage as these proteins interact with both NEDD4 E3 ubiquitin ligases as well as ESCRT proteins and have been shown to influence both multivesicular body formation and viral budding [58]. Under this scenario, phosphorylation of the PPXY motif could regulate DI particle release by modifying the accessibility of the PPXY domain to NEDD4 E3 ubiquitin ligases.

All viruses must strike a balance between pathogenesis and persistence, with the ultimate goal of ensuring their own maintenance in nature. It has long been postulated that DI particles may be one way in which LCMV is able to tip the scales towards persistence, thus lowering its immunological profile and fitness cost to its host. By identifying the specific cellular pathway required to form these DI particles, and the apparent importance of viral phosphorylation in accessing the pathway, our findings raise the possibility that arenaviruses can dynamically adjust DI particle production in response to external environmental factors. Further, the PPXY mutant viruses we have developed represent a new tool that will allow the field to formally test the importance of DI particles for the establishment of persistent LCMV infection in reservoir rodents. The ability of the PPXY late domain to drive the production of DI particles is a novel finding with important implications for understanding host-pathogen relationships and the design of vaccines and antivirals. In particular, PPXY-containing viruses such as Ebola virus and Lassa virus are known to persist for long periods following the resolution of acute human disease [59–61]. It is possible that DI particles play a significant role in allowing these viruses to persist in humans, similar to their presumed importance for infection in reservoir species. Therefore, targeting DI particle formation could be a promising approach to clear persistent infection in humans. Finally, the possibility that the PPXY late domain and ESCRT machinery could broadly drive DI particle production in other virus families represents an exciting area of future research.

## Materials and Methods

### Cells and viruses

Human embryonic kidney cells (HEK-293T/17) (CRL-11268, American Type culture Collection, Manassas, VA) (referred to as HEK293T cells in the manuscript) were maintained in Dulbecco’s Modified Eagle Medium (DMEM) (11965–092) supplemented with 10% fetal bovine serum (FBS) (16140–071), 1% penicillin-streptomycin (15140–122), 1% MEM Non-Essential Amino Acids Solution (11140–050), 1% HEPES Buffer Solution (15630–130), and 1% GlutaMAX (35050–061) purchased from Invitrogen (Carlsbad, CA). L929 mouse fibroblast cells (CCL-1, American Type culture Collection) were maintained in Minimum Essential Medium (MEM) (11095–080) supplemented with 10% FBS, 1% penicillin-streptomycin, 1% MEM Non-Essential Amino Acids Solution, 1% HEPES Buffer Solution, and 1% GlutaMAX. Baby hamster kidney cells (BHK-21) were kindly provided by M. J. Buchmeier (University of California, Irvine) and grown in DMEM supplemented with 10% FBS, 1% penicillin-streptomycin, and 1% GlutaMAX. African green monkey kidney cells (Vero E6) were kindly provided by J. L. Whitton (The Scripps Research Institute, La Jolla) and grown in DMEM supplemented with 10% FBS, 1% penicillin-streptomycin, and 1% HEPES Buffer Solution. T-Rex HEK293 cells stably transduced with a tetracycline-inducible plasmid encoding WT or dominant negative EQ mutant vacuolar protein sorting 4A (VPS4A) or VPS4B as described in [40–42] were generously provided by M. Kielian (Albert Einstein College of Medicine, Bronx) and were maintained in DMEM supplemented with 10% FBS, 1% penicillin-streptomycin, 1% MEM Non-Essential Amino Acids Solution, 1% HEPES Buffer Solution, 1% GlutaMAX, and 100 μg/mL Zeocin (R250-01, Invitrogen). VPS4 expression was induced by incubating cells in the above growth medium containing 1 μg/mL tetracycline as described [42]. All cell lines were grown at 37°C in a humidified incubator containing 5% CO_2_. Lymphocytic choriomeningitis virus (LCMV) strain Armstrong 53b was kindly provided by J. L. Whitton. Wild-type vesicular stomatitis virus expressing green fluorescent protein (VSV-GFP) as described in [62] was kindly provided by J. Hiscott (Vaccine and Gene Therapy Institute of Florida, Port St. Lucie) and M. Shaw (Icahn School of Medicine at Mount Sinai, New York). Junín virus (JUNV) C#1, which is an attenuated vaccine strain derived from the virulent WT JUNV strain XJ as described in [63,64], was kindly provided by M. Buchmeier (University of California, Irvine) and R. Tesh (The University of Texas Medical Branch at Galveston). Working stocks of these viruses were amplified and titered (via plaque assay) on Vero E6 cells. See below under “Generation of Recombinant LCMV” for a description of the recombinant (r)LCMV strain Armstrong 53b that were generated for this study.

### Plasmids

The LCMV Armstrong 53b Z protein (WT, Y88A, Y88E, or Y88F) was subcloned into a modified pCAGGS expression vector [65] and different combinations of these plasmids were used to screen for the phosphorylation of Z (Fig 1D) or the budding efficiency of Z (Fig 2E). The WT and Y88 mutant Z genes were fused to the streptavidin binding peptide (SBP) (MDEKTTGWRGGHVVEGLAGELEQLRARLEHHPQGQREP) through an 18 base pair linker at the C-terminus of Z to permit affinity purification and western blot detection of Z. The nucleotide sequence of the WT Z gene matches NCBI gene identifier number AY847351 while the translated amino acid sequence for the WT Z gene matches Protein Locus number AAX49343. WT Z was amplified by PCR from the pT7-L(+) plasmid generously provided by J. C. de la Torre (The Scripps Research Institute, La Jolla) [66] using the forward primer LCMVZf (5’-ACAAGTTTGTACAAAAAAGCAGGCTGATATCGCCACCATGGGTCAAGGCAAGTCCAGA-3’), which has a 5’ overhang containing Gateway AttB1 and Kozak sequences and the reverse primer LCMVZr (5’-ACCTCCACCTCCAGCTGCCTCTTCGTAGGGAGGTGGAGA-3’), which has an overhang containing the linker sequence. The SBP tag was amplified from the pT7-FLAG-SBP-1 plasmid (P3871, Sigma-Aldrich, St. Louis, MO) via PCR using the forward primer SBPf (5’- GCAGCTGGAGGTGGAGGTATGGACGAAAAAACCACCGGT-3’), which has a 5’ overhang containing the linker sequence, and the reverse primer SBPr (5’-ACCACTTTGTACAAGAAAGCTGGGTCTTACGGTTCACGCTGACCCTGCGG-3’), which contains a 3’ overhang with a stop codon preceding an AttB2 sequence. The two products were fused by PCR using the Z forward primer and the SBPr primer. The cassette was subcloned into the pCAGGS vector using the Gateway cloning system (Invitrogen) following the manufacturer’s instructions as has been previously described [65,67]. The plasmids pC-NP and pC-GP, which express the LCMV Armstrong 53b nucleoprotein (NP) and glycoprotein (GP), respectively, and plasmids pol-I S and pol-I L, which express the LCMV L and S genome segments, respectively, were used to generate rLCMV. These reagents were generously provided by J. C. de la Torre and are described in [68]. Each of the Y88 mutant Z genes used in these studies were synthesized and subcloned into the pCAGGS or pol-I L vectors, respectively, by Biobasic, Inc. (Markham, ON, Canada). A pol-I L vector containing an SBP-tag directly fused to the C-terminus of Z was also generated by Biobasic, Inc. All plasmid sequences were verified by DNA sequencing.

### Identification of phosphorylated residues by mass spectrometry

To identify phosphorylation sites on LCMV Z via mass spectrometry, Vero E6 cells were infected with LCMV strain Armstrong 53b and 48 hr later cell-free virions were purified by sucrose-banding as described previously [69]. Purified virions were then lysed in Triton buffer (0.5% NP40, 1% Triton X-100, 140mM NaCl, and 25mM Tris-HCl containing a protease inhibitor cocktail (04693159001, Roche Applied Science, Indianapolis, IN)) and mixed with Laemmli sample buffer (62.5 mM Tris-HCl, 10% glycerol, 2% sodium dodecyl sulfate and 0.01% bromophenol blue (B392, Fisher Scientific, Pittsburgh, PA)) containing 5% 2-mercaptoethanol. Virion protein lysates were separated on a 4–20% Tris-Glycine polyacrylamide gel (EC60255, Invitrogen). The gel was stained with Coomassie (40% methanol, 20% acetic acid, and 0.1% Brilliant Blue R (B7920, Sigma-Aldrich)), destained with a solution of 30% methanol and 10% acetic acid, and then imaged using a Canon Canoscan 8800F scanner. For mass spectrometry, the protein band corresponding to the Z protein was excised and cut into 1 mm cubes and processed with chemicals from Fisher Scientific as follows. The gel pieces were rinsed with HPLC grade water and then incubated with destain solution (50 mM ammonium bicarbonate and 50% acetonitrile) for 30 minutes at 37°C. The destain solution was removed and the gel pieces were dehydrated by incubating twice with 100% acetonitrile for 5 minutes. The gel pieces were reduced with 25 mM dithiothreitol in 50 mM ammonium bicarbonate for 30 minutes at 55°C. After cooling for 10 minutes at room temperature, the gel pieces were dehydrated by incubating with 100% acetonitrile for 5 minutes and then alkylated in the dark with 10 mM iodoacetamide in 50 mM ammonium biocarbonate for 45 minutes at room temperature. The gel pieces were washed two times in destain solution for 5 minutes, dehydrated with 100% acetonitrile, then rehydrated with water for 10 minutes. The gel pieces were further dehydrated with two 5 minute incubations in 100% acetonitrile before removing all liquid and drying the gel pieces at room temperature for 10 minutes. The gel pieces were rehydrated with a solution of 12.5 ng/μL sequencing grade chymotrypsin (V1061, Promega, Madison, WI) or 12.5 ng/μL sequencing grade modified trypsin (V5111, Promega) in 50 mM ammonium bicarbonate on ice for 30 minutes, before digesting overnight at 37°C. Peptides were extracted with a solution of 2.5% formic acid in 50% acetonitrile while spinning in a microcentrifuge at 13,000 rpm for 10 minutes. The supernatant was removed and saved while the gel pieces were subjected to further extraction and rinsing with 100% acetonitrile. The second extraction was combined with the initial extraction. All solvent was removed from the extracts using a vacuum centrifuge at 37°C. The peptides were resuspended in 2.5% formic acid, 2.5% acetonitrile prior to mass spectrometry analysis. Peptides were separated over 12 cm of Magic C18, 5 μM, 200 Å reversed phase material (PM5/66100/00, Michrom Bioresources, Auburn, CA) in a microcapillary column using a MicroAS autosampler (Thermo Scientific, Pittsburgh, PA). Following 15 minutes of isocratic loading in 2.5% acetonitrile, 0.15% formic acid, the peptides were eluted from the column with a 5–35% gradient of acetonitrile with 0.15% formic acid over 40 minutes using a Surveyor Pump Plus HPLC (Thermo Scientific). Mass spectra were acquired either in an LTQ-XL linear ion trap, or in a linear ion trap-orbitrap mass spectrometer (Thermo Scientific) as described previously [70]. Briefly, for most analyses 10 data-dependent MS/MS spectra followed each survey scan. However, in several cases after obtaining the initial spectra for phosphopeptides we followed up with targeted MS/MS spectra in order to increase fragment ion coverage. The IPI human forward and reverse concatenated database was used to search the raw data using SEQUEST software requiring tryptic peptides and either a 2 Da precursor mass tolerance (for precursor data acquired in the LTQ) or 20 PPM (for precursor data acquired in the orbitrap). In the searches the following precursor mass differences were allowed: serine, threonine, and tyrosine residues (+79.96633 Da); methionine (+15.99492 Da) and cysteines (+57.02146 Da or 71.0371).

### Validation of Z phosphorylation

To confirm that Z was phosphorylated in human cells as well as cells from rodent cells, in Fig 1D and 1E, plasmid-derived Z expressed in HEK293T cells and Z from rLCMV Z-SBP-infected cells were both probed for phosphotyrosine signal via western blot. For plasmid-derived Z, 2 x 10^5^ HEK293T cells were seeded in a 12-well plate and transfected the next day with 0.8 μg per well of pLCMV-Z WT, pLCMV-Z Y88F, or an empty vector using 0.8 μL of a 1 mg/mL solution of polyethylenimine (23966, Polysciences, Inc., Warrington, PA) per well. For Z derived from rLCMV Z-SBP-infected cells, 2.5 x 10^5^ L929 cells were seeded in 6-well plates and infected the next day at an MOI of 0.01. Two days following the transfection or infection, H_2_0_2_ at a final concentration of 8.8 mM or an equivalent volume of H_2_0 was spiked into the appropriate wells containing HEK293T or L929 growth media. After a 15 minute incubation, the cells were lysed in Triton buffer containing a protease inhibitor cocktail and PhosStop phosphatase inhibitor cocktail (04906837001, Roche Applied Science) and the SBP-tagged Z proteins were affinity purified using magnetic streptavidin beads as previous described [67]. The purified proteins were separated via SDS-PAGE and screened for Z or tyrosine phosphorylated-Z via standard chemiluminescent western blotting and detected with film (Fig 1D) or with a LI-COR C-Digit digital imager (LI-COR, Lincoln, NE) (Fig 1E).

### Generation of recombinant (r)LCMV

rLCMV WT, rLCMV Z-SBP and rLCMV containing Z-Y88 mutations (Y88F, Y88E, Y88A) were generated using the previously described reverse genetics system [68]. Briefly, 10 μL of Lipofectamine 2000 (52887, Invitrogen) was mixed with 100 μL of OptiMEM (31985, Invitrogen) and then added to a plasmid mixture consisting of 1.6 μg pC-NP, 2.0 μg pC-L, 1.6 μg pol-I S, and 2.8 μg pol-I L (WT, Z-SBP or containing the described Y88 point mutations) in 100 μL OptiMEM and incubated at room temperature for 25 minutes. 200 μL of this transfection mixture and 800 μL of OptiMEM was then added to 1 well of a 6-well plate which had been seeded the previous day with 3.5 x 10^5^ BHK-21 cells and washed prior to transfection with 1 mL of OptiMEM. The cells were incubated with the transfection mixture for 4 hr after which the media was replaced with BHK-21 growth media diluted 5-fold in DMEM. Three days later the supernatant was collected, clarified by centrifugation at 1,200 RPM for 5 minutes at 4°C, and used to infect a fresh monolayer of 1.8 x 10^6^ BHK-21 cells in a T-75 flask. Following a 1 hr absorption, the inoculum was removed and fresh BHK-21 growth media diluted 5-fold in DMEM was added to the cells. Three days later the supernatant of this flask was collected, clarified by centrifugation, and titered by plaque assay. To generate an expanded virus stock, Vero E6 cells were infected with this material at an MOI of 0.0001 and 48 or 72 hr later, supernatants were collected, clarified, and titered by plaque assay. A portion of the L segment (most of the Z gene, the intergenic region, and part of the L gene) of each rLCMV Y88 mutant was sequenced to ensure that these viruses had not reverted. The material used for this sequencing was derived from the 72 hr pi time point shown in Fig 2B. Viral RNA from clarified supernatants was isolated using the Qiagen Viral RNA mini kit (52906, Qiagen, Valencia, CA) according to the manufacturer’s protocol. Viral RNA was converted to cDNA using primer L 845- (5’- GCAGGACTTGAGGGCTATGA-3’), Superscript III (18080–044, Invitrogen), RNAse Out (10777–019, Invitrogen), and 5 μL of RNA following the manufacturer’s protocol for first strand cDNA synthesis. A portion of the L-segment containing Z was amplified with 30–40 cycles of PCR using Platinum *Pfx* DNA polymerase (11708–013, Invitrogen) and primers L126+ (5’- ATAGTACAAACAGGGCCGAAATCC-3’) and L764- (5’- TTTGTTGGGTTCAGAGATAAGTGT-3’) following the manufacturer’s protocol. The PCR product was prepared for sequencing using ExoSAP-IT (78200, Affymetrix, Santa Clara, CA) following the manufacturer’s protocol and sequenced by the University of Vermont Cancer Center DNA Analysis Facility.

### SDS-PAGE and western blotting

Protein lysates were diluted in Laemmli sample buffer containing 5% 2-mercaptoethanol and separated on NuPAGE 4–12% Bis-Tris gels with MES buffer. Protein was transferred to nitrocellulose membranes using iBlot gel transfer stacks (IB301001 or IB301002, Invitrogen) and the Invitrogen iBlot Device as directed by the manufacturer. Efficient protein transfer was confirmed by staining membranes with a solution containing 0.1% Ponceau S (P3504, Sigma-Aldrich) and 5% acetic acid which was subsequently removed by washing with water. Two methods were used for protein detection: quantitative LI-COR-based detection or standard chemiluminescent-based detection. For quantitative LI-COR analysis, membranes were blocked with a solution of 5% milk in PBS for 1 hr and incubated overnight at room temperature with the indicated primary antibodies diluted in PBS containing 5% milk and 0.2% Tween 20 (BP337, Fisher Scientific). Following 5 washes in PBS with 0.5% IGEPAL CA-630 (198596, MP Biomedicals, Solon, OH), the membranes were incubated for 1 hr at room temperature with secondary antibodies diluted in PBS containing 5% milk, 0.2% Tween 20 and 0.02% sodium dodecyl sulfate, washed 5 times in PBS with 0.5% IGEPAL CA-630 and 1 time with PBS, then imaged using the LI-COR Odyssey CLx imaging system. For quantitative LI-COR analysis of VPS4B in Figs 6A, 6B and S5, membranes were probed using the iBind Flex western device (SLF2000, Thermo Scientific) with the iBind Flex fluorescent detection solution kit (SLF2019, Thermo Scientific) following the manufacturer’s instructions. For chemiluminescent-based detection of phosphorylated proteins, the same general procedure was used with the following exceptions: i) membranes were blocked with either PBS containing 5% milk and 0.05% IGEPAL CA-630 or protein-free blocking buffer (37572, Thermo Scientific), ii) primary and secondary antibodies were diluted in PBS containing 5% milk, 0.05% IGEPAL CA-630, and 3% fetal bovine serum or protein-free blocking buffer, and iii) the secondary antibodies were incubated with the membrane for 2 hr.

The following primary antibodies were used for western blotting (at the indicated concentrations): mouse anti-streptavidin binding peptide (MAB10764, Millipore, Billerica, MA) (1:10,000), rabbit anti-actin (A2066, Sigma-Aldrich) (1:10,000), mouse anti-actin (A5441, Sigma-Aldrich) (1:5,000), rabbit anti-actin (A2066, Sigma-Aldrich) (1:2,500), mouse anti-phosphotyrosine (clone 4G10, Millipore) (0.2 μg/mL), mouse anti-green fluorescent protein (632380, Clontech, Mountain View, CA) (1:1,000), rabbit anti-LCMV Z (880) (1:500), mouse anti-LCMV GP2 (33.6) (1:2,000), and rabbit anti-LCMV nucleoprotein (2165) (1:5,000). Antibodies 880, 2165, and 33.6 were generously provided by M. J. Buchmeier (University of California, Irvine). For quantitative western blotting, the following secondary antibodies from LI-COR were used: IRDye 800CW goat anti-mouse (926–32210) for the Z release assay in Fig 2E at 1:20,000 and in Figs 6A, 6B and S5 at 1:3,000 (for probing by iBind) and IRDye 680LT goat anti-mouse (926–68020) and IRDye 800CW Goat anti-rabbit (926–32210) were used at 1:20,000 to detect proteins in Fig 3A–3D. IRDye 680LT goat anti-mouse was used at 1:3,000 (for probing by iBind) in S5 Fig to detect actin. A horseradish peroxidase-conjugated anti-mouse secondary antibody (71045, EMD Millipore, Billerica, MA) diluted 1:5,000 was used for chemiluminescent-based detection in Fig 1D and 1E.

### Virus growth curve

To determine the growth kinetics of rLCMV in Fig 2B, 6-well plates were seeded with 1.9 x 10^5^ Vero E6 cells per well. The following day the cells were infected with each respective rLCMV at an MOI of 0.01. Supernatants were collected at 12, 24, 36, 48, and 72 hr pi, clarified by centrifugation at 1,200 RPM for 5 minutes at 4°C, then titered by plaque assay.

### Z-virus-like particle (VLP) release assay

To determine the release efficiency of the Y88 mutant Z proteins in Figs 2E and S5, 2 x 10^5^ HEK293T cells or T-Rex HEK293 cells stably transduced with a tetracycline-inducible plasmid encoding WT or dominant negative EQ mutant VPS4B were seeded in a 12-well plate. The next day cells were transfected with 0.8 μg per well of pLCMV-Z WT, Z-G2A, -Z Y88F, -Z Y88E, or -Z Y88A using 0.8 μL of a 1 mg/mL solution of polyethylenimine per well. For the experiments shown in S5 Fig, VPS4B expression was induced with 1 μg/mL tetracycline at the time of transfection. The following day (24 hr post-transfection) cells and VLP-containing media (which had been clarified) were collected, lysed with Triton lysis buffer, and subjected to quantitative western blotting. For detection of Z from VLPs produced in VPS4B WT or DN cell lines, SBP-tagged Z was affinity purified from lysed VLP-containing media using magnetic streptavidin beads prior to quantitative western blot analysis. To calculate the percent VLP release we first normalized each Z protein value (from supernatants or cells) by the sum of all Z protein bands on a particular gel as described in [71]. The percent VLP release was then calculated as the quotient of the Z protein quantity in VLPs divided by the quantity of Z in cells [(Z_mut_VLP / Z_mut_cells) / (Z_WT_ VLP/ Z_WT_ cells)].

### Plaque assay and measurement of plaque size and cytopathic effect

To measure infectious virus titers, a standard plaque assay was employed as follows. Six-well plates were seeded with 1 x 10^5^ (LCMV and JUNV) or 1 x 10^6^ (VSV) Vero E6 cells per well and the following day inoculated with 10-fold serial dilutions of virus in a total volume of 0.5 mL of Vero E6 growth medium. Following a 90 minute absorption at 37°C, the cells were overlaid with a solution of 0.7% agarose (20–102, Apex Industrial Chemicals, Aberdeen, United Kingdom) in Vero E6 growth media. The plates were fixed 2 (VSV) or 4 (LCMV and JUNV) days later with a solution of 2.5% formaldehyde (1635-4L, Sigma) in 3x PBS. Following removal of the agarose plugs, the fixed monolayers were stained with 0.1% crystal violet (C581-100, Fisher Scientific) and 2.1% ethanol in water. To determine the plaque size of rLCMV in Fig 2D or the overall level of cytopathic effect induced by these viruses in Figs 4A, 4B, 6C and 6D, the wells were imaged with an Alpha Innotech digital camera paired to a Computar H6Z0812M motorized zoom lens. The area of each plaque as well as the mean pixel intensity of each well was calculated using ImageJ software.

### Plaque interference assay

To determine the titer of LCMV DI particles, samples were transferred to clear snap cap tubes (21-402-904, Thermo Scientific) and irradiated for 2 minutes with UV light in a UVP CL-1000 ultraviolet crosslinker in to kill standard infectious virus. The samples were serially diluted in 5-fold increments and added to 24-well plates which had been seeded the previous day with 20,000 (LCMV and JUNV C#1) or 100,000 (VSV) Vero E6 cells per well. Subsequently, 50 PFU per well of rLCMV WT (or 50 PFU per well of JUNV C#1 or VSV in Fig 5A) was added to each well containing UV-irradiated samples. UV-irradiated samples were also added to a second set of wells to which no standard virus was added to ensure that all infectious virus had been eliminated from the samples. After a 90 minute absorption period at 37°C, the cells were overlaid with a solution of 0.7% agarose in Vero growth media and left at 37°C. The plates were fixed and stained 2 (VSV) or 4 (LCMV and JUNV C#1) days later as above for the plaque assay. The plaques were counted in each well and the plaque interfering unit 50 (PIU_50_) was calculated using the plaque reduction statistical web tool (https://exon.niaid.nih.gov/plaquereduction). Because a unique biochemical or genetic signature to differentiate standard infectious virus particles from DI particles has not been defined, the assay we employed relied on measurement of the interfering activity of DI particles as opposed to a direct physical measure of the particles themselves. For Fig 5B, rLCMV WT was filtered with either 0.45 μM (28145–481, VWR, Radnor, PA) or 0.2 μM (09-719C, Fisher Scientific) syringe filters or Amicon 30K (UFC903024, Millipore) or 10K (UFC901024, Millipore) centrifugal filters prior to treatment with UV light and DI titering as above.

### Virus challenge in inducible VPS4A- and VPS4B-expressing cell lines

To determine the role of the ESCRT pathway in LCMV release, 2.5 x 10^5^ T-Rex HEK293 cells stably transduced with a tetracycline-inducible VPS4A or VPS4B (WT or dominant negative EQ in each case) were seeded in 6-well plates that were first coated with poly D-lysine (P6407, Sigma-Aldrich) for 5 minutes then washed 3x with PBS. Cells were infected 24 hr later with rLCMV WT at an MOI of 0.001. Forty-eight hr later (when all cells were productively infected) the cells were induced with growth medium containing 1 μg/mL tetracycline or a medium only control. Six hr after induction cells were washed 3x with PBS and fresh growth medium containing 1 μg/mL tetracycline or medium alone were added. Eighteen hr later the cells and supernatants were collected. In Fig 6A and 6B, the cell lysates were probed for VPS4B DN or WT protein (via the GFP fusion tag on these proteins) or actin expression by quantitative western blotting. Supernatants were titered by plaque assay for infectious virus and DI particle levels by measuring the cytopathic effect in a plaque assay with equal PFUs of virus in each well (as described under plaque assay) and/or by plaque interference assay. The role of VPS4B in VSV release was also tested. For the VSV challenge studies, 5 x 10^5^ VPS4B WT or EQ cells were seeded in poly D-lysine treated wells and 24 hr later treated with either growth medium containing 1 μg/mL tetracycline or medium alone. One hr later, the cells were infected with VSV at an MOI of 10. One hr following infection, the cells were washed 3x with PBS and fresh growth medium containing 1 μg/mL tetracycline or medium alone was added. Six hr later the cells and supernatants were collected and assessed by quantitative western blotting and plaque assays, respectively.

In order to verify uniform VPS4B expression as well as rLCMV WT infection by microscopy, in parallel to the experiment described above, 5 x 10^4^ cells were seeded on poly D-lysine-treated 12mm glass coverslips in 24-well plates. At the time of harvest (24 hr post-infection) the coverslips were rinsed with PBS, fixed with 4% paraformaldehyde (15714, Electron Microscopy Sciences, Hatfield, PA) in PBS for 20 minutes, then washed 2x with PBS for 5 minutes. The cells were permeabilized with 0.1% Triton X-100 in 1% bovine serum albumin (BSA) in PBS, blocked with 10% normal goat serum (005-000-121, Jackson, West Grove, PA) in 1% BSA in PBS, and immunostained with anti-LCMV nucleoprotein antibody (1.3–3) (1:500) kindly provided by M. Buchmeier (University of California, Irvine) and secondary anti-mouse Alexafluor 555 (A28180, Thermo Scientific) (1:1,000) each for 1 hr in 1% BSA in PBS. DNA was detected with 4’, 6-diamidino-2-phenylindole hydrochloride (DAPI) (D9542, Sigma Aldrich) in 1% BSA in PBS. Cells were washed with 1% BSA in PBS in between each step. Images were acquired on a Zeiss LSM 510 laser scanning confocal microscope using a 63X objective lens. Post-capture image processing was carried out in FIJI and Photoshop; the GFP fluorescence, NP staining, and DAPI signal are shown at equal exposures in all conditions.

### Virion concentration and fractionation

To determine the NP, GP, and Z protein content of rLCMV virions in Fig 3A–3D, 2 x 10^6^ Vero E6 cells were seeded in a T-150 culture flask and infected the next day at an MOI of 0.01, 0.001, or 0.0001. At 48 or 72 hr following inoculation, the supernatant was collected, clarified by centrifugation, and screened for infectious virus by plaque assay. An equal number of plaque forming units of each virus (range 1 to 3x10^7^ PFU per experiment) were layered onto a solution of 20% sucrose in TNE buffer, pH 7.4 (10 mM Tris base, 1 mM EDTA, 0.2 M NaCl) and centrifuged for 2 hr at 30,000 rpm at 4°C in a Thermo-Scientific Sorval WX Ultra 80 ultra centrifuge equipped with a Sorval Surespin 630 rotor. The resulting virus pellet was resuspended in 2X-concentrated Laemmli buffer containing 5% 2-mercaptoethanol, then analyzed by SDS-PAGE and quantitative western blotting.

To separate rLCMV by gradient centrifugation in S2 Fig, 2 x 10^6^ Vero E6 cells were seeded in a T-150 culture flask and infected the next day at an MOI of 0.0001. At 72 hr following inoculation, the supernatant was collected and clarified by centrifugation. The clarified supernatants were added to 50 mL tubes (430290, Corning) containing polyethylene glycol (PEG) 8000 (81268, Sigma-Aldrich) and sodium chloride such that the final concentrations were 10% and 1%, respectively. The solutions were incubated at 4°C on a rotating platform for 2 hr then were centrifuged for 30 minutes at 10,000 rpm at 4°C in a Thermo-Scientific Sorval Legend RT+ centrifuge equipped with a Sorval Fiberlite F15-8x50cy rotor. The supernatant was removed and the virus-PEG pellet was resuspended in TNE buffer and screened for infectious virus by plaque assay. Density gradients were prepared by layering solutions of 7%, 10%, 13%, 16%, and 19% optiprep (D1556, Sigma-Aldrich) diluted in PBS in 36 mL tubes (03141, Thermo Scientific) then leaving overnight at 4°C to allow a continuous gradient to form. An equal number of plaque forming units of each virus (range 4 x 10^7^ to 1 x 10^8^ PFU per experiment) was layered onto the continuous gradient and centrifuged for 12 hr at 30,000 rpm at 4°C in a Thermo-Scientific Sorval WX Ultra 80 ultracentrifuge equipped with a Sorval Surespin 630 rotor. The resulting separated virus was collected in 2 mL fractions using a New Era NE-9000G programmable peristaltic pump and titered via plaque assay.

### Quantitative RT-PCR

To enumerate copies of LCMV S and L segment genomic RNA contained in virions for Fig 3E and 3F, viral RNA was extracted from cell-free virions using the Qiagen Viral RNA mini kit according to the manufacturer’s instructions and subjected to quantitative RT-PCR as previous described [72]. Briefly, cDNA was generated in a 50 μL RT reaction containing 5 μL of viral RNA, 0.2 μM of the gene specific primer S 2865- (5’-CAGGGTGCAAGTGGTGTGGTAAGA-3’) or L 5906- (5’- TGGGACTGAGTTTCGAGCATTACG-3’), which are complementary to the S or L segment genomic RNA, 5 μL of 10x PCR Buffer II (#E12874, Applied Biosystems, Carlsbad, CA), 5 μL of 10 mM dNTP mix (362275, Applied Biosystems), 1 μL RNase inhibitor (N808-0119, Applied Biosystems), and 1.25 μL of Multiscribe reverse transcriptase (4308228, Applied Biosystems). RT reaction conditions were 25°C for 10 minutes, 48°C for 30 minutes, and 95°C for 5 minutes. Quantitative PCR was then performed in a 25 μL reaction volume consisting of 5 μL of cDNA, 0.9 μM each of the forward primer S 2275+ (5’-CGCTGGCCTGGGTGAAT-3’) or L 5517+ (5’-GGCCTTGTATGGAGTAGCACCTT-3’) and reverse primer S 2338- (5’-ATGGGAAAACACAACAATTGATCTC-3’) or L 5645- (5’-GGTCTGTGAGATATCAAGTGGTAGAATG-3’), 0.2 μM of the TaqMan probe S 2295+ (5’-6FAM-CTGCAGGTTTCTCGC-MGBNFQ-3’) or L 5582- (5’-6FAM-CTGAAGAATACCACCTATTATACCA-MGBNFQ-3’), and 12.5 μL of the TaqMan Universal PCR Master Mix (4326614, Life Technologies, Grand Island, NY). Reaction conditions were 95°C for 10 minutes followed by 40 cycles of 95°C for 15 seconds and 60°C for 1 minute. Copy numbers of LCMV S or L segment genomic RNAs were calculated by comparison with a series of standard dilutions of the pT7-S or pT7-L plasmids as described [72]. Data was generated on an Applied Biosystems StepOnePlus Real-Time PCR System and analyzed with the provided software.

### Statistical analysis

Statistical analysis was performed using GraphPad Prism software. For the virus growth curves in Fig 2B, the data was first log transformed, then a two-way analysis of variance (ANOVA) was performed with a Holm-Sidak’s test for multiple comparisons to compare viruses at each time point. A one-way ANOVA with Holm-Sidak’s test for multiple comparisons was used to analyze the VLP release assay in Fig 2E, the viral protein levels in concentrated virions in Fig 3B–3D, and the S and L segment to PFU ratios in Fig 3E and 3F. To compare plaque area in Fig 2D, the data were first tested for normality using the D’Agostino and Pearson omnibus normality test, then the Kruskal-Wallis non-parametric test was used and multiple comparisons were made with Dunn’s multiple comparisons test. To analyze the cytopathic effect induced by rLCMV WT or Z-Y88 mutants (Fig 4B) or by rLCMV WT generated in VPS4B WT or dominant negative cells (Fig 6D), a two-way ANOVA was performed with the Holm-Sidak’s test for multiple comparisons. To compare VSV or LCMV virus titers, LCMV DI particle titers, or Z VLP levels produced in VPS4B WT or EQ cells (Fig 6A, 6B and 6E and S5 Fig) a two-tailed unpaired t test with Welch’s correction was performed. To compare DI particle titers in Fig 5A–5C, a value of 19 PIU_50_/mL (just below the limit of detection value of 20 PIU_50_/mL) was substituted for samples that were below the limit of detection and then a one way ANOVA was performed. For all statistical analyses, the data utilized was generated from at least 3 independent experiments as indicated in each respective figure legend.

## Supporting Information

S1 Fig Fragment ion tables from mass spectra and spectral counts of phosphorylated and unphosphorylated tryptic peptides. (A-B) For the indicated phosphorylated (A) and unphosphorylated (B) peptides, corresponding to the spectra shown in Fig 1B, the calculated and measured (colored numbers) *m/z* values of the y- and b-type ions are shown. (C) The phosphorylated and unphosphorylated peptides detected from virion-derived LCMV Z in Fig 1A are listed. Each MS/MS spectrum was manually examined and found to be correct by a comparison to spectra with the highest Xcorr values and by comparing predicted and observed fragment ions. (A and C) Y^#^ indicates phosphorylated tyrosine.
(TIF)

S2 Fig Profile of standard infectious rLCMV WT or Y88 particles following separation via density ultracentrifugation. Vero E6 cells were infected with rLCMV WT, Y88F, Y88E, or Y88A at an MOI of 0.0001 and 72 hr later supernatants were clarified, precipitated with PEG-8000, resuspended in TNE, and titered for PFU via plaque assay. An equal number of PFU for each rLCMV was layered onto an optiprep gradient (7%, 10%, 13%, 16%, and 19%) and centrifuged for 12 hr at 30,000 RPM at 4°C. The entire gradient was collected in 15 fractions of 2 mL each. Each fraction was titered for PFU via plaque assay. Shown are results from 2 independent experiments.
(TIF)

S3 Fig Efficient DI particle formation requires a functional ESCRT pathway. (A-C) T-Rex HEK293 cells stably transduced with vectors for tetracycline-based induction of WT vacuolar protein sorting 4A (VPS4A) or the DN VPS4A mutant, EQ, were infected with rLCMV WT and 2 d later treated with tetracycline to induce the expression of WT or DN VPS4A. 6 hr after VPS4A induction (54 hr pi), the cells were washed and given fresh media containing tetracycline. Supernatants were collected 18 hr later (72 hr pi) and titered via plaque assay. The results shown in (A) represent the mean PFU ± SEM from 2 independent experiments that contained 3 technical replicates and were tested for statistical significance with an unpaired t test with Welch’s correction. Equivalent PFUs of virus (range 2 x 10^1^ to 2 x 10^3^) produced from WT or DN VPS4A cells were inoculated onto monolayers of Vero E6 cells and a standard plaque assay was performed. Representative images of crystal violet-stained wells are shown in (B). Inhibition of standard infectious virus-induced cytopathic effect by DI particles at each dose was determined in (C) by measurement of the mean pixel intensity of each well using Image J software. The data in (C) are representative of the mean ± SEM relative to WT VSP4A (at 200 PFU per well) from 2 independent experiments that contained 3 technical replicates and were tested for statistical significance with a two way ANOVA and Holm-Sidak’s test for multiple comparisons. (C) *p < 0.05, as determined by the indicated statistical tests.
(TIF)

S4 Fig LCMV generates more DI particles per standard infectious particle than JUNV C#1. Serial 10-fold dilutions of stock preparations of LCMV or JUNV C#1 were inoculated onto monolayers of Vero E6 cells and a standard plaque assay was performed to visualize DI-mediated interference of standard virus at low dilutions.
(TIF)

S5 Fig Expression of dominant negative VPS4B does not impact the ability of LCMV Z to form VLPs. (A-B) T-Rex HEK293 cells stably transduced with a tetracycline-inducible plasmid encoding WT or dominant negative EQ mutant vacuolar protein sorting 4B (VPS4B) were simultaneously transfected with a plasmid encoding LCMV Z WT and exposed to tetracycline to drive the expression of WT or DN VPS4B. One day later both the cells and VLP-containing supernatants were collected. Z from VLP-containing supernatants was affinity purified with magnetic streptavidin beads. The quantity of Z affinity purified from VLPs or present in the corresponding whole cell lysates was determined via quantitative western blotting. The percent VLP release shown in (A) was calculated as the amount of Z protein found in the cell culture media relative to the amount in cells. Data are presented as mean release ± SEM relative to WT Z from 3 independent experiments. A one way ANOVA with Holm-Sidak’s test for multiple comparisons was used to compare the mean values. n.s., not significant). In panel (B), cell lysates were also screened by western blotting to verify the induction of VPS4B WT or EQ expression using an anti-GFP antibody and for actin as a loading control.
(TIF)
