# Supplementary figures and images for: The Lymphocytic Choriomeningitis Virus Matrix Protein PPXY Late Domain Drives the Production of Defective Interfering Particles
Source: PLoS Pathog. 2016 Mar 24;12(3):e1005501. doi: 10.1371/journal.ppat.1005501 (PMC4806877; doi:10.1371/journal.ppat.1005501)

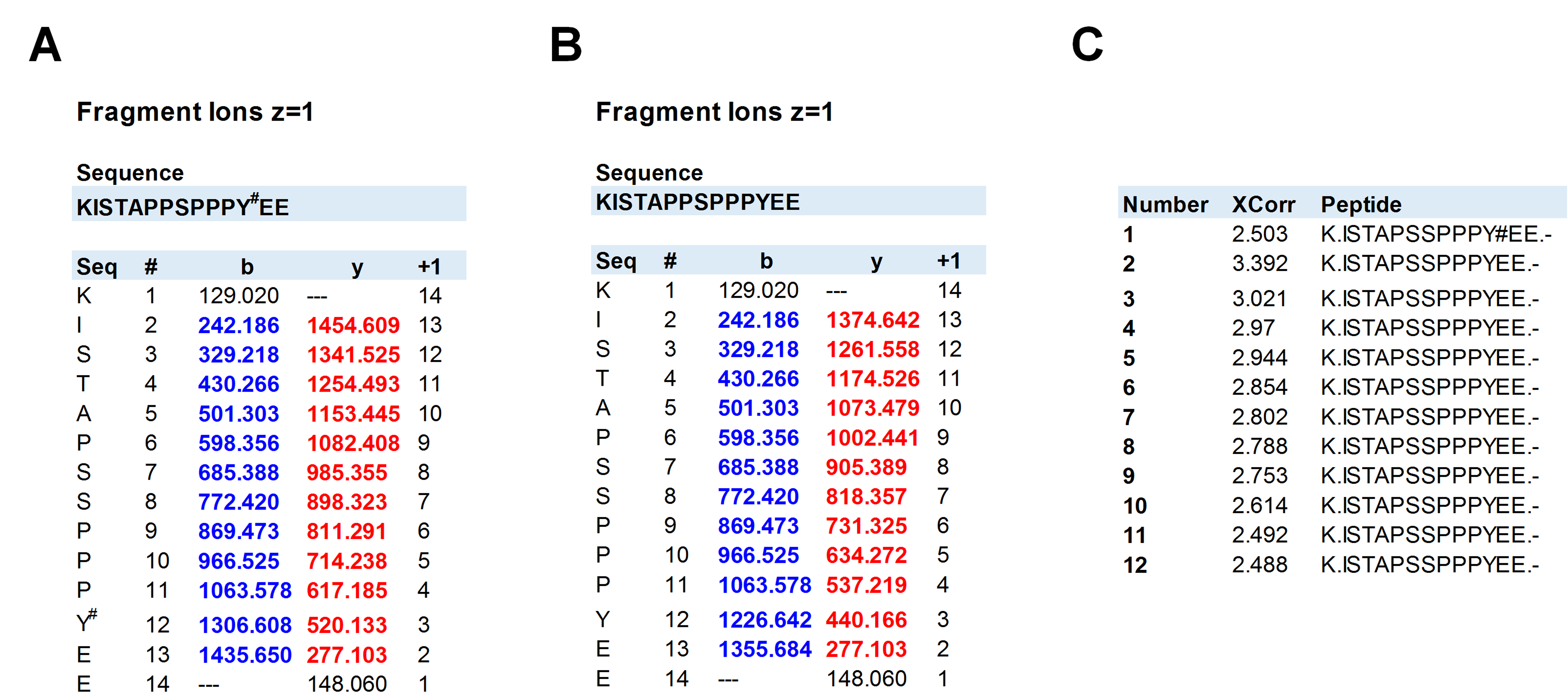

Supplement: S1 Fig — (A-B) For the indicated phosphorylated (A) and unphosphorylated (B) peptides, corresponding to the spectra shown in Fig 1B, the calculated and measured (colored numbers) m/z values of the y- and b-type ions are shown. (C) The phosphorylated and unphosphorylated peptides detected from virion-derived LCMV Z in Fig 1A are listed. Each MS/MS spectrum was manually examined and found to be correct by a comparison to spectra with the highest Xcorr values and by comparing predicted and observed fragment ions. (A and C) Y# indicates phosphorylated tyrosine. (TIF) [file ppat.1005501.s001.tif]

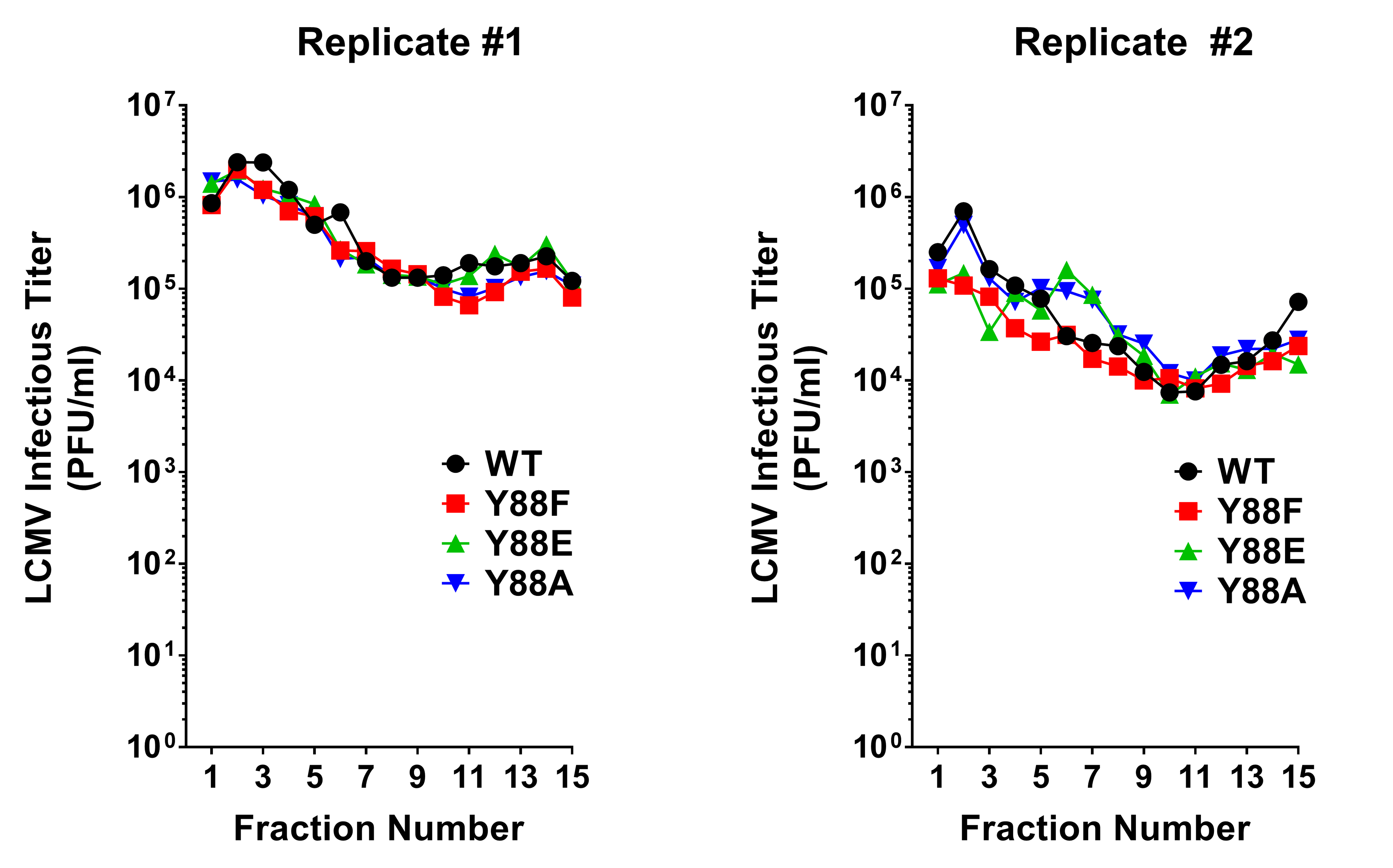

Supplement: S2 Fig — Vero E6 cells were infected with rLCMV WT, Y88F, Y88E, or Y88A at an MOI of 0.0001 and 72 hr later supernatants were clarified, precipitated with PEG-8000, resuspended in TNE, and titered for PFU via plaque assay. An equal number of PFU for each rLCMV was layered onto an optiprep gradient (7%, 10%, 13%, 16%, and 19%) and centrifuged for 12 hr at 30,000 RPM at 4°C. The entire gradient was collected in 15 fractions of 2 mL each. Each fraction was titered for PFU via plaque assay. Shown are results from 2 independent experiments. (TIF) [file ppat.1005501.s002.tif]

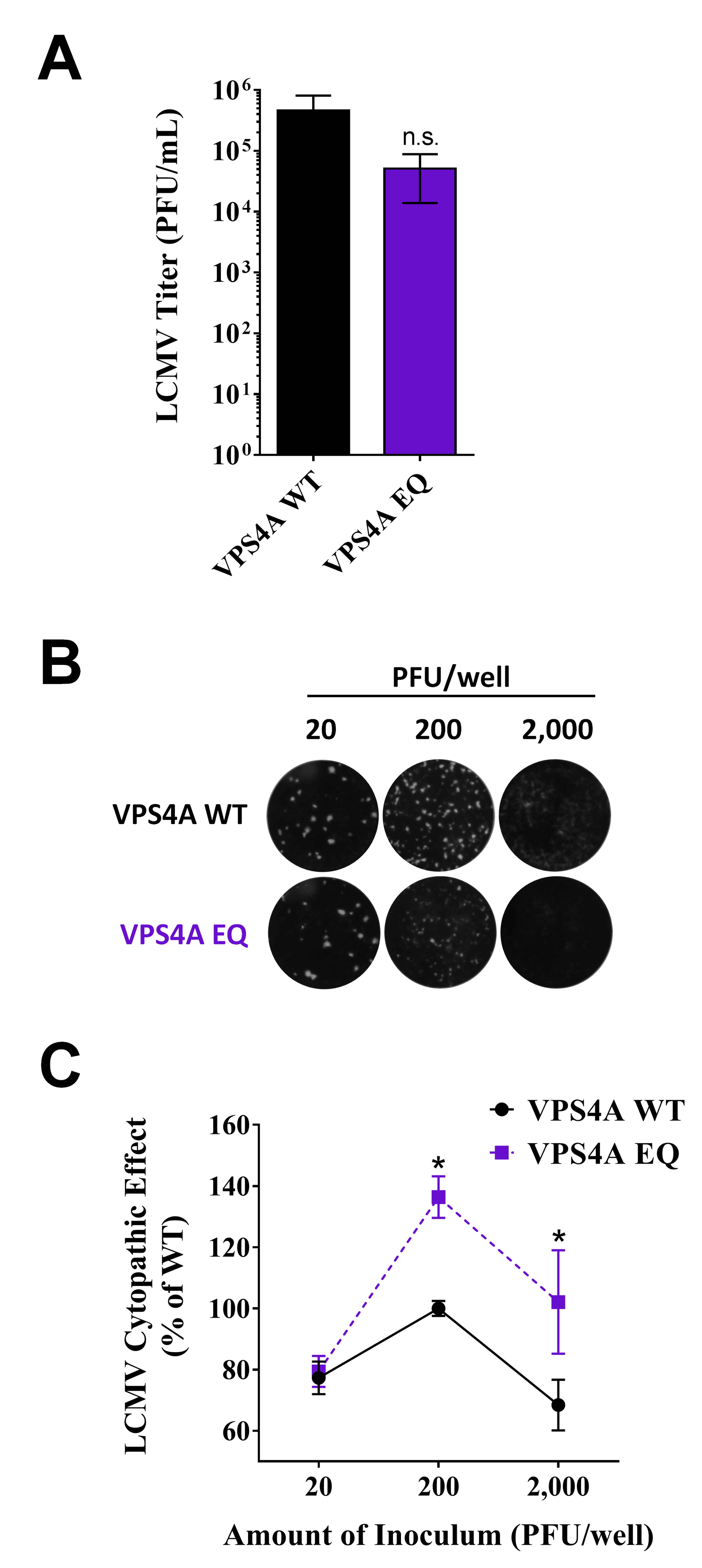

Supplement: S3 Fig — (A-C) T-Rex HEK293 cells stably transduced with vectors for tetracycline-based induction of WT vacuolar protein sorting 4A (VPS4A) or the DN VPS4A mutant, EQ, were infected with rLCMV WT and 2 d later treated with tetracycline to induce the expression of WT or DN VPS4A. 6 hr after VPS4A induction (54 hr pi), the cells were washed and given fresh media containing tetracycline. Supernatants were collected 18 hr later (72 hr pi) and titered via plaque assay. The results shown in (A) represent the mean PFU ± SEM from 2 independent experiments that contained 3 technical replicates and were tested for statistical significance with an unpaired t test with Welch’s correction. Equivalent PFUs of virus (range 2 x 101 to 2 x 10^3^) produced from WT or DN VPS4A cells were inoculated onto monolayers of Vero E6 cells and a standard plaque assay was performed. Representative images of crystal violet-stained wells are shown in (B). Inhibition of standard infectious virus-induced cytopathic effect by DI particles at each dose was determined in (C) by measurement of the mean pixel intensity of each well using Image J software. The data in (C) are representative of the mean ± SEM relative to WT VSP4A (at 200 PFU per well) from 2 independent experiments that contained 3 technical replicates and were tested for statistical significance with a two way ANOVA and Holm-Sidak’s test for multiple comparisons. (C) *p < 0.05, as determined by the indicated statistical tests. (TIF) [file ppat.1005501.s003.tif]

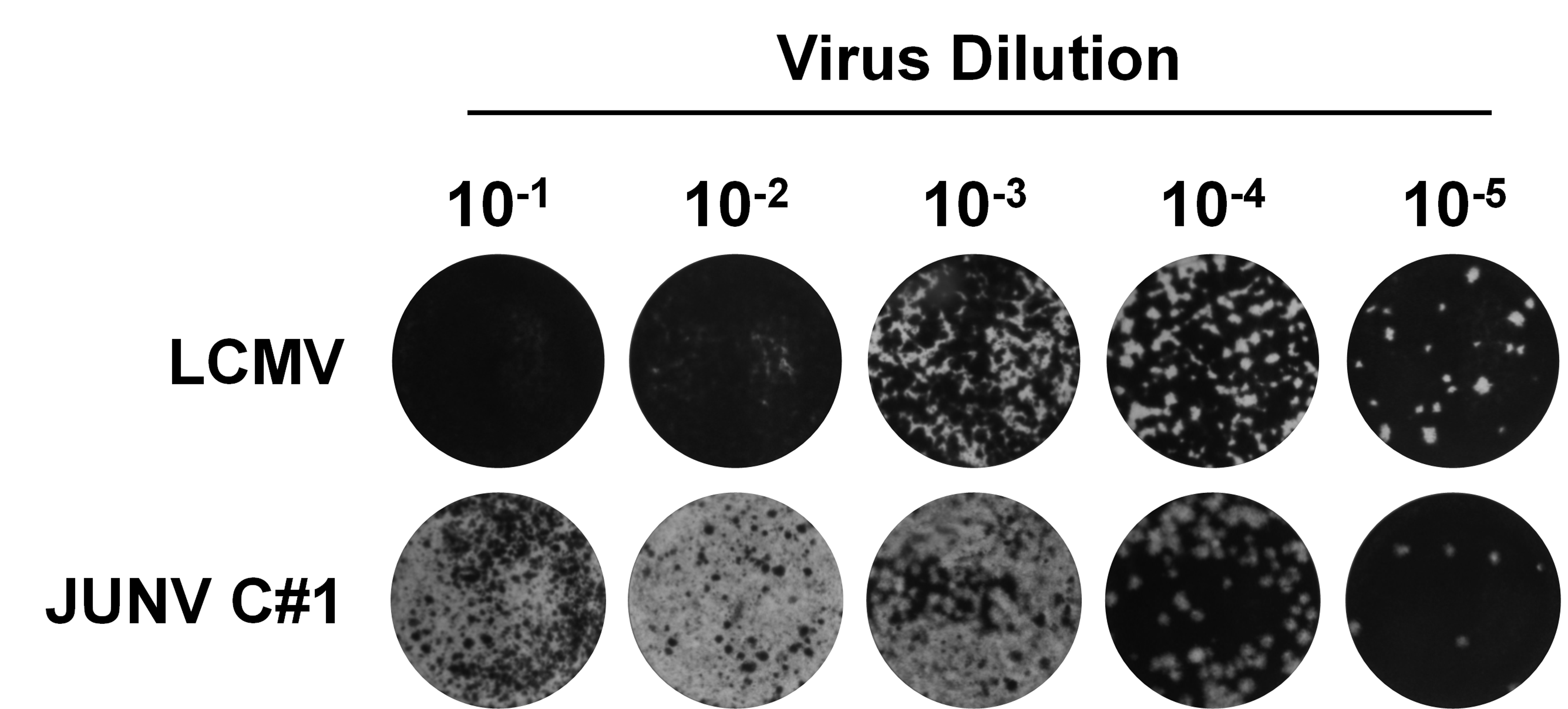

Supplement: S4 Fig — Serial 10-fold dilutions of stock preparations of LCMV or JUNV C#1 were inoculated onto monolayers of Vero E6 cells and a standard plaque assay was performed to visualize DI-mediated interference of standard virus at low dilutions. (TIF) [file ppat.1005501.s004.tif]

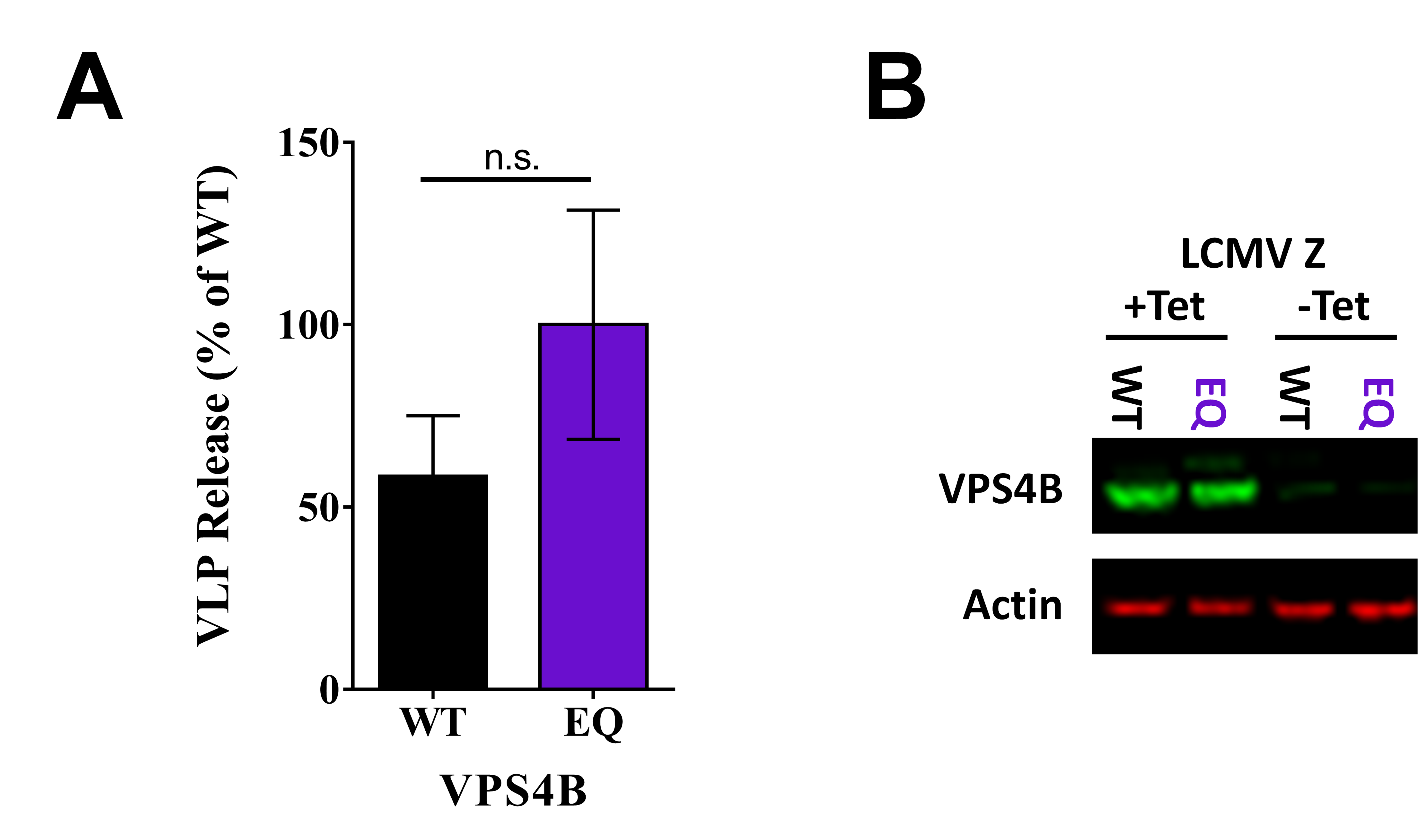

Supplement: S5 Fig — (A-B) T-Rex HEK293 cells stably transduced with a tetracycline-inducible plasmid encoding WT or dominant negative EQ mutant vacuolar protein sorting 4B (VPS4B) were simultaneously transfected with a plasmid encoding LCMV Z WT and exposed to tetracycline to drive the expression of WT or DN VPS4B. One day later both the cells and VLP-containing supernatants were collected. Z from VLP-containing supernatants was affinity purified with magnetic streptavidin beads. The quantity of Z affinity purified from VLPs or present in the corresponding whole cell lysates was determined via quantitative western blotting. The percent VLP release shown in (A) was calculated as the amount of Z protein found in the cell culture media relative to the amount in cells. Data are presented as mean release ± SEM relative to WT Z from 3 independent experiments. A one way ANOVA with Holm-Sidak’s test for multiple comparisons was used to compare the mean values. n.s., not significant). In panel (B), cell lysates were also screened by western blotting to verify the induction of VPS4B WT or EQ expression using an anti-GFP antibody and for actin as a loading control. (TIF) [file ppat.1005501.s005.tif]
